# Iteratively Refined Guide Trees Help Improving Alignment and Phylogenetic Inference in the Mushroom Family *Bolbitiaceae*

**DOI:** 10.1371/journal.pone.0056143

**Published:** 2013-02-13

**Authors:** Annamária Tóth, Anton Hausknecht, Irmgard Krisai-Greilhuber, Tamás Papp, Csaba Vágvölgyi, László G. Nagy

**Affiliations:** 1 Department of Microbiology, Faculty of Science and Informatics, University of Szeged, Szeged, Hungary; 2 Department of Systematic and Evolutionary Botany, Faculty Centre of Biodiversity, University of Vienna, Wien, Austria; Montreal Botanical Garden, Canada

## Abstract

Reconciling traditional classifications, morphology, and the phylogenetic relationships of brown-spored agaric mushrooms has proven difficult in many groups, due to extensive convergence in morphological features. Here, we address the monophyly of the Bolbitiaceae, a family with over 700 described species and examine the higher-level relationships within the family using a newly constructed multilocus dataset (ITS, nrLSU rDNA and EF1-alpha). We tested whether the fast-evolving Internal Transcribed Spacer (ITS) sequences can be accurately aligned across the family, by comparing the outcome of two iterative alignment refining approaches (an automated and a manual) and various indel-treatment strategies. We used PRANK to align sequences in both cases. Our results suggest that – although PRANK successfully evades overmatching of gapped sites, referred previously to as alignment overmatching – it infers an unrealistically high number of indel events with natively generated guide-trees. This 'alignment undermatching' could be avoided by using more rigorous (e.g. ML) guide trees. The trees inferred in this study support the monophyly of the core Bolbitiaceae, with the exclusion of *Panaeolus*, *Agrocybe*, and some of the genera formerly placed in the family. *Bolbitius* and *Conocybe* were found monophyletic, however, *Pholiotina* and *Galerella* require redefinition. The phylogeny revealed that stipe coverage type is a poor predictor of phylogenetic relationships, indicating the need for a revision of the intrageneric relationships within *Conocybe*.

## Introduction

### The Family Bolbitiaceae

Brown-spored mushroom genera of the Bolbitiaceae represent members of a large, complex clade in the Agaricales with a considerable history of taxonomic debate. They live as decomposers of leaf-litter and dung and are characterized mostly by tiny fruiting bodies and a cap covering composed of balloon-shaped cells [1], [2]. Some of the species contain hallucinogenic compounds and are toxic [3]. Traditionally the genera *Bolbitius, Conocybe, Agrocybe, Galerella, Pholiotina, Descolea, Panaeolus, Panaeolina* and a number of smaller genera have been placed in the family [2], [4], [5], [6], [7], [8]. In addition, a number of puffball-like (gasteroid) species have been added, including *Gastrocybe, Galeropsis, Agrogaster, Gymnoglossum* and *Cyttarophyllum*, many of which are known only from type materials [2], [9], [10]. *Agrocybe* has traditionally been classified in the Bolbitiaceae, which has been abandoned by many authors recently [4], [11], [12]. This was in part inspired by hitherto published molecular phylogenies, which included only a handful of species from the Bolbitiaceae, but suggested a polyphyletic origin, with *Agrocybe* being distantly related to the rest of the genera [3], [13], [14], [15], [16], [17]. This has also been supported by patterns of conidiogenesis [11].

*Panaeolus*, *Panaeolopsis* and *Panaeolina* form a rather isolated group in the Bolbitiaceae. They share several taxonomically important features with the core Bolbitiaceae (*Bolbitius, Conocybe* and *Pholiotina*), such as structure of spore wall and cap covering, or ecology, but differ in a number of spectacular features [2]. Among others, the color of the spores is dark reddish-brown to blackish in the panaeoloid species, whereas the bolbitioid genera have yellow- to rusty brown spores. A close affinity between *Panaeolus* and *Panaeolina* and *Conocybe*/*Bolbitius* has been inferred previously [14], [16] mainly based on nrLSU sequences, however, support for this relationship, and thus the correct phylogenetic classification of the panaeoloid genera remained elusive. The only available multigene datasets, however, suggest that the panaeoloid species are more closely related to species of *Tubaria* and allied genera than the core Bolbitiaceae [17], [18].

*Bolbitius, Conocybe* and *Pholiotina* have emerged as a monophyletic unit in almost all phylogenetic studies involving these species with limited taxon sampling [13], [14], [16], [17]. The generic-level taxonomy of *Pholiotina* and *Conocybe* has been a disputed field. Several authors treated *Pholiotina* as a subgenus (e.g. [7]) within *Conocybe*. The latter is characterized by special capitate cystidia (lecythiform), which clearly separates it from all but two of the *Pholiotina* taxa (*Ph. brunnea, Ph. intermedia*), bearing fusiform - utriform cystidia. Furthermore, many species of *Pholiotina* possess a protective veil coverage of the young fruiting bodies, while species of *Conocybe* and some *Pholiotina* species do not [1], [4], [5]. This puts the monophyly of this genus into question and raises the possibility of a relationship between veil-less *Pholiotina* and *Conocybe* taxa. Despite this long-standing debate, no molecular phylogenetic studies have resolved the affinities of *Conocybe* and *Pholiotina* or veiled and veil-less species of *Pholiotina* so far.

**Figure 1:**
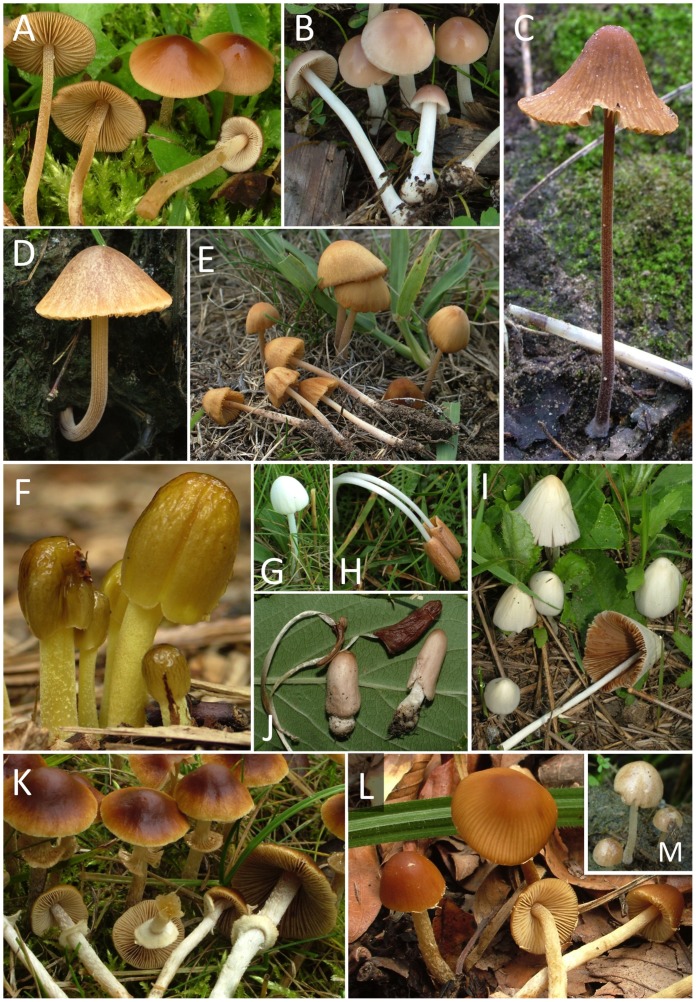
Morphological diversity in the Bolbitiaceae, with examples of *Conocybe* (A–E, G–J), *Bolbitius* (F) and *Pholiotina* (K–M). A. *Conocybe mesospora*; B. *Conocybe hornana*; C. *Conocybe digitalina;* D. *Conocybe anthracophila*; E. *Conocybe microrhiza*; F. *Bolbitius titubans*; G. *Conocybe romagnesii* a partially deliquescent species; H and J *Conocybe deliquescens* upper fresh, lower immature and already collapsed fruiting bodies; I. *Conocybe albipes*; K. *Pholiotina teneroides* showing rich veil remnants on stipe; L. *Pholiotina brunnea* with scanty veil fibrils on cap margin; M. *Pholiotina coprophila*, a species without veil.

The largest genus in the family is *Conocybe*, with about 500 described species, which, according to a recent critical revision [5], group into ca. 170 morphologically distinguishable taxa (see Fig. 1). Sectional taxonomy of *Conocybe* predominantly relies on the composition of stipe covering, made up of two types of cystidia, which serve to protect the stipe in early stages of the ontogeny and prevent the fusion of gill edges with the stipe surface when the cap is still closed [2], [4], [5], [6], [7]. These include oil-flask-shaped, lecythiform cystidia, i.e. cells with a broad base and sharply delimited rounded capitulum, and simple hair-like or cylindrical cystidia. The three largest sections of the *Conocybe*, sect. *Conocybe*, sect. *Pilosellae* and sect. *Mixtae* have lecythiform, hair-like and both types of cystidia on the stipe, respectively [4], [12]. Section *Mixtae* was erected for species with both lecythiform and hair-like cystidia occurring on the stipe [19]. However, because of the occasional occurrence of distorted or defective cystidia among lecythiform ones, this definition was later emended to comprise only species in which the ratio of lecythiform and hair-like cystidia ranges from 1∶5 to 5∶1 [20], [21]. Based on different types of characters, several morphologically uniform groups have been excepted from this classification, such as section *Candidae* for species with pseudoparaphyses (spacer cells between basidia), pale colours of the fruiting bodies and (partial) deliquescence, section *Ochromarasmius* for species with ornamented spores, or section *Singerella* for volvate taxa [5], [7], [22], [23]. The presence of pseudoparaphyses led some authors to consider species of section *Candidae* as members of *Bolbitius* [24]. Despite the mentioned exceptions, whether the traditional view of three major sections can be validated with molecular characters remained untested.

### The Evolution of Fruiting Bodies within and Outside of the Bolbitiaceae

The Bolbitiaceae include species with deliquescent fruiting bodies, named after a special phase of the ontogeny, in which the fruiting body is enzymatically digested, resulting in partial autolysis or collapse [2], [25], [26], [27]. Recently, in the Psathyrellaceae, deliquescent fruiting bodies have been identified as a result of convergent, complex evolutionary processes involving correlated gain of several physiological traits. The term coprinoidization was proposed to describe this process, as an analogue of gastromycetation [26], [28]. It has been suggested that deliquescent fruiting bodies represent an adaptation to fast-changing environments, which is achieved by an accelerated ontogeny resulting from quick water uptake of pseudoparaphyses, cystidia and skeletal cells of the cap. Previous phylogenetic results highlighted the polyphyletic nature of deliquescent fruiting bodies at the family level by identifying *Coprinus* s. str. in the Agaricaceae [29], and the remaining *Coprinus* sensu lato taxa in the Psathyrellaceae, however, whether deliquescent species were monophyletic within these families remained contentious. Recently the situation has been resolved in the Psathyrellaceae by identifying four lineages with independent acquisitions of deliquescent fruiting bodies [28]. Whether a similar scenario of fruiting body evolution with similar putative adaptations can be discerned in the Bolbitiaceae remained to be tested in order to obtain a more thorough picture on fruiting body evolution in mushrooms.

### Alignment Methods for Indel-rich Sequences

Aligning rapidly evolving and indel-rich loci across higher evolutionary distances has been a challenge for evolutionary biologists and considerable effort has been put into developing increasingly sophisticated methods that capture more of the biological realism [30], [31], [32], [33], [34], [35], [36], [37]. The accurate inference of the number of insertions and deletions (indels) and their placement in the alignment has been the task of primary interest, since most of the rapidly evolving phylogenetic markers are also non-coding and thus accumulate indels at a high rate [36], [38]. Recent advances in probabilistic models eliminated the problem of gap costs, i.e. penalizing indels rather than incorporating them into alignment inference [34], [36], [39]. For instance, PRANK achieved theoretical superiority over traditional methods with gap costs, by using an explicit model of indel evolution and distinguishing insertions from deletions [33]. Even with elaborate nucleic acid and indel models, the dependence of progressive algorithms on a guide tree can induce significant bias and should be accounted for in downstream analyses. It has been recognized rather early that rough guide trees built from pairwise or k-mer distances often poorly reflect true relationships and can bias the progressive alignment steps of the algorithms [30]. One approach to mitigate the dependence on a rough guide tree has been the application of few to several iterative refinement steps during alignment estimation, where each step a new alignment is generated, which serves as the input for the estimation of a new guide tree (e.g. [30]). A potential drawback of these algorithms is the poor quality of the guide tree, which is usually built by simple distance-based algorithms. Liu et al. [31], [32] automated the process of alignment estimation and the inference of high quality trees (by Maximum Likelihood) in an algorithm that iteratively improves the alignment. This method, called SATé, uses the likelihood of the resulting ML trees as an optimality criterion for choosing among alignments [32] and has been shown to achieve higher accuracy at a not significantly higher computational cost. SATé uses the whole alignment in the tree inference stage, thus the potential confounding effects of poorly aligned sites may be carried over to the next cycle. Recently, using PRANK, a manual alternative for this has been proposed, which takes advantage only of unambiguously aligned regions [40]. A tree is computed from ungapped (unambiguously aligned) regions of the alignment and used as a guide tree in the next round of alignment estimation. However, the way alignment noise affects the quality of guide trees and the next alignment has not been examined.

In the present study, we examine the relationship between alignments and guide trees by applying a manual iterative method to improve the alignments of indel-rich loci and eliminate guide-tree induced errors based on phylogenetic signal from other genes. We do this by using a three-locus dataset of the Bolbitiaceae, a fungal family for which practically no phylogenetic information has been available so far. We used the above alignment strategy to address the alignability of the rapidly evolving nuclear ITS region, which is the most frequently sequenced marker in fungi and plants, as well as two additional genes (nrLSU and EF1-alpha), which generally pose no alignment problems. We demonstrate that the ITS region can be reliably aligned at this scale, by testing for conflict between single-gene trees as well as the ITS alignment with gapped regions removed by an automatic method. The resulting multigene phylogeny is then used to study the evolution of fruiting body types in the Bolbitiaceae and to confirm large-scale patterns of fruiting bodies described recently in other families.

## Materials and Methods

### Taxon Sampling

To obtain a comprehensive view of the phylogenetic relationships within the Bolbitiaceae, we sampled a morphologically diverse set of species from all major groups of the family. Thus, we gathered specimens of the genera *Conocybe, Pholiotina, Bolbitius, Descolea, Galerella* and *Tubariella*, and selected ca. 70% of the species accepted in a recent critical monographic treatment of the genera, covering both temperate and tropical species (Table 1). *Psathyrella michiganensis*, a species morphologically fitting *Conocybe* has also been included in the study [41]. Within *Conocybe*, we selected taxa so as to maximize the morphological diversity in the sample, by sampling species from each of the sections recently established [4], [5]. The final data set contained sequences of 123 specimens of 116 species of *Conocybe* (91 taxa), *Bolbitius* (6), *Pholiotina* (13), *Descolea* (1), *Galerella* (2), *Panaeolus* (2) and *Galeropsis* (1). All species were represented by at least two genes (except *C. volviornata*). Altogether 118 ITS, 114 LSU and 87 EF1-alpha sequences have been produced for this study. PCR or sequencing of *Galerella plicatella* (WU20898), *Descolea phlebophora* (WU27464), *D. recedens* (WU27465), *D. sp.* (WU27469) and *Tubariella rhizophora* (WU22233) failed, so these species have been omitted from the analyses. The newly generated sequences have been complemented with ITS and LSU sequences of *Bolbitius psittacinus* (EF648217, EF648218), *Panaeolus sphinctrinus* (DQ182503, DQ470817) and *Panaeolus cambodginiensis* (AB158633). Based on the ML phylogeny of species of the agaricoid clade, we chose *Panaeolus* as the outgroup for the Bolbitiaceae.

**Table 1:** List of specimens, voucher numbers, countries of origin and GenBank accession numbers of the species used in this study.

			Accession Numbers		
Taxon name	Voucher No.	Country of Origin	ITS	LSU	ef–1a
*Bolbitius coprophilus*	SZMC-NL-2460	Hungary	DQ234567	DQ234567	DQ234567
*Bolbitius elegans*	WU23943	Italy	JX968250	JX968367	JX968456
*Bolbitius lacteus*	WU8327	Austria	JX968224	JX968342	–
*Bolbitius reticulatus*	WU30001	Hungary	JX968249	JX968366	JX968455
*Bolbitius subvolvatus*	WU28379	Italy	JX968248	JX968365	JX968454
*Bolbitius vitellinus*	SZMC-NL-1994	Hungary	JX968252	JX968369	–
*Conocybe aff. ochrostriata*	SZMC-NL-0830	Hungary	JX968236	JX968354	JX968447
*Conocybe alboradicans*	SZMC-NL-3226	Hungary	JX968219	JX968336	JX968435
*Conocybe alboradicans*	WU14678	Austria	JX968220	JX968337	–
*Conocybe ammophila*	WU23983	Mongolia	JX968197	JX968313	JX968416
*Conocybe anthracophila var. ovispora*	WU25461	Italy	JX968237	JX968355	–
*Conocybe anthracophila*	WU14367	Italy	JX968212	JX968329	JX968430
*Conocybe antipus*	WU19791	Austria	JX968215	JX968332	JX968432
*Conocybe aurea*	WU28161	Italy	JX968184	JX968300	JX968407
*Conocybe bispora*	SZMC-NL-2573	Hungary	JX968203	JX968320	JX968423
*Conocybe brachypodii*	SZMC-NL-2105	Sweden	JX968191	JX968307	JX968413
*Conocybe brachypodii*	SZMC-NL-2189	Hungary	–	JX968314	JX968417
*Conocybe brachypodii*	SZMC-NL-2289	Sweden	JX968183	JX968299	JX968406
*Conocybe brachypodii*	SZMC-NL-3169	Hungary	JX968199	JX968316	JX968419
*Conocybe cettoiana*	WU10436	Italy	JX968218	JX968335	–
*Conocybe crispella*	WU27367	Australia	JX968208	JX968325	JX968426
*Conocybe cylindracea*	WU20796	Italy	JX968240	JX968358	JX968449
*Conocybe deliquescens*	SZMC-NL-0574	Hungary	JX968210	JX968327	JX968428
*Conocybe dumetorum*	SZMC-NL-2693	Sweden	JX968201	JX968318	JX968421
*Conocybe dunensis*	WU27359	Spain	JX968227	JX968345	–
*Conocybe echinata*	SZMC-NL-1007	Hungary	JX968196	JX968312	–
*Conocybe elegans*	SZMC-NL-0908	Sweden	JX968223	JX968341	JX968437
*Conocybe enderlei*	SZMC-NL-0165	Sweden	JX968161	JX968277	JX968389
*Conocybe enderlei*	WU21272	Italy	JX968163	JX968279	–
*Conocybe farinacea*	SZMC-NL-2173	Hungary	JX968167	JX968283	–
*Conocybe fiorii*	WU17793	Italy	JX968217	JX968334	JX968434
*Conocybe fuscimarginata*	SZMC-NL-3668	Sweden	JX968238	JX968356	JX968448
*Conocybe gigasperma*	SZMC-NL-3972	Slovakia	JX968179	JX968295	JX968403
*Conocybe gracilis*	WU21277	Austria	JX968221	JX968338	–
*Conocybe graminis*	WU13466	Austria	JX968195	JX968311	–
*Conocybe herbarum*	WU22193	Austria	JX968193	JX968309	–
*Conocybe hornana*	SZMC-NL-3499	Slovakia	JX968178	JX968294	JX968402
*Conocybe incarnata*	WU21897	Finland	JX968229	JX968347	JX968441
*Conocybe ingridiae*	WU28158	Italy	JX968244	JX968361	JX968451
*Conocybe inocybeoides*	SZMC-NL-3589	Hungary	JX968202	JX968319	JX968422
*Conocybe inopinata*	WU27544	Italy	JX968165	JX968281	JX968392
*Conocybe intrusa*	WU25546	Finland	JX968211	JX968328	JX968429
*Conocybe juniana var. sordescens*	SZMC-NL-2304	Sweden	JX968192	JX968308	JX968414
*Conocybe karinae*	WU28526	Germany	JX968151	JX968268	JX968384
*Conocybe lactea*	SZMC-NL-1012	Hungary	JX968209	JX968326	JX968427
*Conocybe lenticulospora*	SZMC-NL-0923	Sweden	JX968242	JX968359	JX968450
*Conocybe leporina*	SZMC-NL-2380	Hungary	JX968177	JX968293	JX968401
*Conocybe lobauensis*	WU17826	Italy	JX968176	JX968292	JX968400
*Conocybe macrocephala*	WU18148	Austria	JX968182	JX968298	–
*Conocybe macrospora*	WU17030	Germany	JX968175	JX968291	–
*Conocybe merdaria*	WU25359	Austria	JX968174	JX968290	–
*Conocybe microrhiza*	SZMC-NL-2180	Hungary	JX968222	JX968340	JX968436
*Conocybe microspora*	SZMC-NL-1890	Hungary	JX968160	JX968276	–
*Conocybe monicae*	WU22612	Austria	JX968200	JX968317	JX968420
*Conocybe moseri var bisporigera*	SZMC-NL-1904	Hungary	JX968235	JX968353	JX968446
*Conocybe nigrescens*	WU27557	Italy	JX968234	JX968352	JX968445
*Conocybe ochrostriata var. favrei*	WU29786	Italy	JX968245	JX968362	JX968452
*Conocybe pallidospora*	WU17079	USA	JX968158	–	–
*Conocybe pallidospora*	WU7395	Austria	JX968239	JX968357	–
*Conocybe papillata*	SZMC-NL-2370	Hungary	JX968216	JX968333	JX968433
*Conocybe pilosella*	SZMC-NL-0831	Hungary	JX968231	JX968349	JX968443
*Conocybe pseudocrispa*	WU18009	Austria	JX968230	JX968348	JX968442
*Conocybe pubescens*	SZMC-NL-1986	Romania	JX968173	JX968289	JX968399
*Conocybe pubescens*	WU20759	Italy	JX968170	JX968286	JX968396
*Conocybe rickeniana*	SZMC-NL-2468	Hungary	JX968198	JX968315	JX968418
*Conocybe romagnesii*	WU26605	Italy	JX968206	JX968323	JX968424
*Conocybe rostellata*	SZMC-NL-2499	Sweden	JX968162	JX968278	JX968390
*Conocybe sabulicola*	WU11185	Italy	JX968186	JX968302	JX968409
*Conocybe semiglobata 'type affinis'*	WU8794	Austria	JX968188	JX968304	JX968168
*Conocybe semiglobata*	SZMC-NL-1993	Hungary	JX968181	JX968297	JX968405
*Conocybe semiglobata var campanulata*	SZMC-NL-3159	Sweden	JX968284	–	JX968394
*Conocybe semiglobata var campanulata*	WU26395	Germany	JX968169	JX968285	JX968395
*Conocybe siennophylla*	SZMC-NL-1210	Hungary	JX968246	JX968363	JX968453
*Conocybe siennophylla*	WU17988	Germany	JX968243	JX968360	–
*Conocybe siliginea*	SZMC-NL-1211	Hungary	JX968159	JX968275	–
*Conocybe siliginea*	SZMC-NL-2313	Sweden	JX968225	JX968343	JX968438
*Conocybe singeriana*	WU22129	Austria	JX968166	JX968282	JX968393
*Conocybe solitaria*	WU20903	India	JX968214	JX968331	JX968431
*Conocybe sp.*	SZMC-NL-1455	Hungary	JX968194	JX968310	JX968415
*Conocybe subovalis*	SZMC-NL-1415	Hungary	JX968190	JX968306	JX968412
*Conocybe subpubescens*	SZMC-NL-0162	Sweden	JX968189	JX968305	JX968411
*Conocybe subpubescens*	SZMC-NL-2181	Hungary	JX968171	JX968287	JX968397
*Conocybe subxerophytica*	SZMC-NL-0164	Sweden	JX968187	JX968303	JX968410
*Conocybe tenera*	SZMC-NL-	Hungary	JX968185	JX968301	JX968408
*Conocybe tenera*	SZMC-NL-1615	Hungary	JX968180	JX968296	JX968404
*Conocybe tetrasporoides*	WU17385	New Zealand	JX968232	JX968350	–
*Conocybe tuxlaensis*	SZMC-NL-1897	Hungary	JX968164	JX968280	JX968391
*Conocybe vaginata*	WU25703	Sri Lanka	JX968204	JX968321	–
*Conocybe velutinomarginata*	WU28695	Germany	JX968226	JX968344	JX968439
*Conocybe velutipes*	SZMC-NL-2187	Hungary	JX968228	JX968346	JX968440
*Conocybe velutipes var. nitrophila*	WU20916	India	JX968233	JX968351	JX968444
*Conocybe volvata*	WU20900	India	JX968205	JX968322	–
*Conocybe volviornata*	WU22218	Indonesia	–	JX968339	–
*Conocybe watlingii*	WU22744	Finland	JX968172	JX968288	JX968398
*Conocybe zeylandica*	WU20185	La Réunion	JX968207	JX968324	JX968425
*Conocybe zuccherellii*	WU12421	Italy	JX968213	JX968330	–
*Descolea maculata var. occidentalis*	WU21819	Portugal	JX968155	JX968272	–
*Galerella floriformis*	WU22833	Vanuatu	JX968254	JX968371	JX968458
*Galerella nigeriensis*	CNF1/5859	Nigeria	JX968251	JX968368	JX968457
*Galeropsis desertorum*	SZMC-NL-1863	Hungary	JX968154	JX968271	JX968387
*Pholiotina aberrans*	SZMC-NL-3161	Sweden	JX968256	JX968373	JX968459
*Pholiotina aeruginosa*	WU27104	Germany	JX968247	JX968364	–
*Pholiotina aporos*	SZMC-NL-1241	Hungary	JX968260	JX968376	JX968462
*Pholiotina arrheni*	SZMC-NL-2509	Sweden	JX968261	JX968377	–
*Pholiotina brunneola*	SZMC-NL-1216	Hungary	JX968259	JX968375	JX968461
*Pholiotina coprophila*	SZMC-NL-2176	Hungary	JX968156	JX968273	–
*Pholiotina cyanopus*	WU2134	Austria	JX968157	JX968274	JX968388
*Pholiotina dasypus*	SZMC-NL-2279	Hungary	JX968152	JX968269	JX968385
*Pholiotina indica*	WU20891	India	JX968263	JX968378	JX968464
*Pholiotina nemoralis var. dentatomarginata*	SZMC-NL-2921	Hungary	JX968258	JX968374	JX968460
*Pholiotina nemoralis var. dentatomarginata*	SZMC-NL-2962	Hungary	JX968257	–	–
*Conocybe pygmaeoaffinis*	WU16600	Austria	JX968149	JX968382	–
*Pholiotina striipes*	WU26997	Austria	JX968150	JX968267	JX968383
*Pholiotina sulcata*	SZMC-NL-1975	Hungary	JX968153	JX968270	JX968386
*Pholiotina teneroides*	SZMC-NL-3501	Slovakia	JX968264	JX968379	JX968465
*Pholiotina utricystidiata*	WU20164	Germany	JX968262	JX968463	–
*Pholiotina vestita*	SZMC-NL-2191	Hungary	JX968266	JX968381	JX968467
*Pholiotina vexans*	SZMC-NL-3967	Slovakia	JX968265	JX968380	JX968466
*Psathyrella michiganensis*	SMITH 10920 TYPE	USA	JX968241	–	–

### Laboratory Protocols

Genomic DNA was extracted from 2–10 mg of dried herbarium specimens, using the DNeasy Plant Mini Kit (QIAGEN) according to the manufacturer’s instructions. We amplified the ITS1-5.8S-ITS2 (ca. 700 bp), nrLSU (ca. 1500 bp) and the EF1-alpha (ca. 1200 bp) regions using the primer combinations ITS1/4, LROR/LR7 and 983F/2218R, respectively [28]. Amplification protocols and PCR conditions were as described previously [28]: 95 C° for 5 min, 95 C° for 0.5 min, 48–52 C° for 0.5 min, 72 C° for 0.3 min, repeated for 30 cycles, and a final extension at 72° for 4 min. Cleaning and sequencing of PCR products was performed commercially by LGC Genomics (Berlin). Single reads were assembled to contigs by the PreGap and Gap4 programs of the Staden package [42].

### Alignment Strategy

Previous studies revealed that alignment errors can have profound effects on phylogeny reconstruction [58], [59], especially in the case of indel-rich alignments [40]. Of the markers used in this study, the ITS is the most prone to such bias, due to the high number of insertion-deletion events in the ITS1 and ITS2 loci (but not in the 5.8S gene), whereas alignment of the two other loci is straightforward at this taxonomic scale. These length mutations cause most of the problems when aligning distantly related ITS sequences. In order to examine the effects of alignment assumptions on the resulting phylogeny, we applied two different alignment strategies for the ITS locus. The two approaches were common in iteratively improving the alignment based on new guide trees estimated from the results of the previous alignment. However, the first strategy, performed in SATé 1.4 ([32], settings: 100 replicates, aligner: PRANK, tree estimator: RAxML, alignment merger:OPAL, model: GTR+G, other options left at default) used only the ITS alignment as an input for estimating a new guide tree, whereas the second one employed information from all three genes to infer a tree that serves as the guide tree in the next alignment inference stage. This latter was performed manually by first inferring an alignment for the ITS sequences in PRANK ([34], using the +F option to fix already inferred indels, otherwise as default), then merging the resulting ITS alignment with the nrLSU and EF1-alpha alignments, and running a Bayesian MCMC analysis on the combined matrix (see below). The 50% majority rule consensus tree computed on the basis of the Bayesian run (excluding burn-in) was then used as input for the next alignment of ITS sequences. Polytomies of the consensus tree were randomly resolved to zero-length branches in Mesquite [43]. This procedure was repeated until no change to the resulting Bayesian consensus tree topology was observed (see below).

### Combinability Tests

We used the congruence of single-gene ML trees as a criterion for detecting incongruence between the single-gene trees. We performed a Maximum Likelihood bootstrap analysis for each single-locus alignment (as described below). Incongruent, strongly supported (70% or greater) nodes were regarded as a signature of significant conflict.

### Phylogenetic Analyses

We estimated phylogenetic relationships and support values using the final concatenated alignment (ITS, LSU and EF1-alpha) using Bayesian MCMC and ML bootstrapping. Best-fit models of evolution were selected for each locus using the AIC_c_ criterion in jModelTest [44]. The proportion of invariant sites (“I”) was omitted from all models, since this accounts for the same phenomenon as the gamma distribution, and convergence problems in Bayesian analyses have been identified when the two were applied simultaneously.

For the final Bayesian analyses, we coded all indels in the ITS alignment as a separate partition of binary presence/absence characters following Simmons and Ochoterena [45]. The simple indel coding algorithm [45] considers all contiguous sets of gap characters as one single evolutionary event, as opposed to e.g. fifth state coding (used in parsimony) where each gap character is considered a separate event. We checked the congruence of the phylogenetic signal in the gap characters by running a Bayesian MCMC analysis using the indel data only and manually comparing the clade structure to that obtained from nucleic acid data. The model implemented for restriction sites in MrBayes was used for the indel partition with the command "coding = variable" to adjust for constant characters not included. As an alternative to indel-coding, we excluded gapped sites from the ITS alignment by using GBlocks 0.91 [46]. For GBlocks, we used the “less stringent” set of parameters, allowing at most half of the sequences to contain a gap in a single column of the alignment.

Bayesian MCMC analyses were performed in MrBayes 3.1.2 [47] and BEAST 1.6.1. [48]. We ran two replicates of four chains with default priors in MrBayes and three replicates of one chain in BEAST were run for 20.000.000 generations, sampling every 100th tree. The data were divided into ITS1, 5.8S ITS2, nrLSU and EF1-alpha partitions and the parameters of the model were unlinked across partitions. For each partition, we used the GTR+G model. To avoid potential over-partitioning of the dataset, we monitored the posterior distributions and associated parameter variances in Tracer [49] for each partition. High variance and low effective sample sizes were used as signatures of over-partitioning. The burn-in was designated at sufficient topological convergence, as judged by the average standard deviation of split frequencies (<0.01) and the Cumulative and Compare functions of AWTY (*ceb.csit.fsu.edu/* ***awty*** */*) [Wilgenbusch 2004]. The resulting tree samples were used to compute 50% majority rule consensus trees in MrBayes and Sumtrees [50].

Maximum Likelihood inference and bootstrapping was performed in 1000 replicates in RAxML 7.0.4 [51], using the same partitioning scheme as above and the GTRGAMMA model. Bootstrap trees were summarized by the SumTrees script of the Dendropy package.

### Constraint Analyses

The monophyly of volvate species, the genus *Pholiotina*, as well as that of veil-less *Pholiotina* species were tested. For this we inferred ten unconstrained and ten constraint trees in RAxML based on the final concatenated alignment, using the above mentioned settings, and calculated single-site likelihoods for all trees. Constraint trees were constructed manually in Mesquite. Because *Galerella nigeriensis* nested within *Pholiotina*, we constructed the constraint trees allowing this taxon to be resolved outside *Pholiotina*. This resulted in a polytomy, which was resolved according to the ML solution around that node. The CONSEL package was used with default settings to calculate approximately unbiased (AU) test p-values [52].

### Maximum Likelihood Phylogeny of the Agaricoid Clade

We assembled an LSU alignment of the agaricoid clade with the aims to select an outgroup for the Bolbitiaceae and identify the phylogenetic position of deliquescent lineages. For this we downloaded nrLSU sequences (>500 bp) of all species of the agaricoid clade represented in GenBank. Due to inconsistencies in species limits, we did not attempt to reduce this dataset to one sequence per species; however, completely unidentified and environmental sequences were excluded. This dataset included *Cyttarophyllum*, a gasteroid representative of the Bolbitiaceae. An alignment was computed by using MUSCLE [30], followed by minor manual refinement. A ML tree inferred in PhyML 3.0 [53], using the GTR+G model of evolution with 4 rate categories, Subtree Pruning and Regrafting (SPR) as the branch swapping algorithm. As a mean of branch support, we performed approximate Likelihood Ratio Tests, which is a fast alternative of bootstrapping [54]. The aLRT support corresponds to the probability that the branches exist, as compared to the null hypothesis of it having zero-length [54]. A list of sequences included in this alignment is available in the Supplementary Information. The alignment of the entire agaricoid clade contained 1608 nrLSU sequences and 1358 characters.

### Ancestral State Reconstruction

To examine whether coprinoid lineages in the Bolbitiaceae emerged via parallel gains or multiple losses of the coprinoid fruiting body type, we performed ancestral state reconstructions on the most recent common ancestor of the Bolbitiaceae, using the tree obtained from the concatenated data matrix including indels. Fruiting body types were coded as either coprinoid or non-coprinoid. Taxa were coded as coprinoid when their fruiting bodies collapse or deliquesce upon maturing, possess pseudoparaphyses and a plicate cap surface. We coded species with partial deliquescence as coprinoid if they show the above syndrome. This way, we could exclude ambiguities arising from the determination of the level of deliquescence in species with tiny fruiting bodies, which desiccate before autolysis. An Maximum Likelihood approach was chosen and performed with the program BayesTraits 1.0 [55]. We used 1000 phylograms, subsampled by Mesquite from the output of the final Bayesian analysis and reconstructed ancestral states on each tree, by using the 'addmrca' command, which allows topological uncertainty to be taken into account by reconstructing the ancestral state for the least inclusive node which contains all the specified taxa. 25 attempts were made to maximize the likelihood on each tree (mltries = 25). Based on the results of preliminary parsimony mapping, we selected five nodes around which switches in fruiting body type might have occurred (Table 2). A Markov model with two states and no restrictions was applied. Statistical significance was measured by fixing the node of interest in one or the alternative state and comparing the mean of the resulting likelihoods. A difference of two logL unit was taken as evidence for significant support [56]. A fully Bayesian method was also considered, but given the uncertainties in prior optimization, we chose Maximum Likelihood reconstruction over a set of trees.

**Table 2:** Summary of ancestral states of fruiting body types inferred by ML reconstructions in BayesTraits.

Node	Probability of state (0)	–lnL_(0)_	–lnL_(1)_	–(lnL_(1)_+lnL_(0)_)
Node 1 (Bolbitiaceae)	0.937	31.52	33.72	2.205
Node 2 (Bolbitius+Pholiotina 1)	0.763	31.53	33.43	1.9
Node 3 (Bolbitius)	0.311	31.71	32.29	0.59
Node 4 (Conocybe 6)	0.673	31.53	33.54	2.0047
Node 5 (section Candidae)	0.014	34.59	31.54	3.0419

See Fig. 3 for the position of nodes on the phylogeny. State 0 and 1 re present the non-coprinoid and coprinoid fruiting body morphologies, respectively.

Stipe covering was also scored for all taxa in the tree, either as consisting entirely of lecythiform (oil-flask-shaped), fusiform-utriform or both types of cystidia. Since there are three major types of stipe covering, we tested both additive binary coding and multistate coding. Additive binary coding comprised two characters, the presence or absence of hair-like, and that of lecythiform cystidia. Under multistate coding we distinguished 'hair-like only' (state 0), 'hairs and lecythiform' (state 1) and 'lecythiform only' (state 2) conditions. We reconstructed ancestral states for the most recent common ancestor of *Conocybe*. Information on the mean number and types of state changes was obtained by the ‘Summarize state changes on trees’ command in Mesquite 3.0 [43]. 50 mappings were performed on each tree.

## Results

### Alignment

Alignment of the nrLSU and EF1-alpha genes was straightforward, neither contained indels. After trimming non-overlapping leading and trailing gaps, the nrLSU and EF1-alpha alignments were and 1303 and 1179 bp long, respectively. The EF1-alpha alignment contained three introns (positions 1–90, 756–813 and 956–1016), which could not be aligned unambiguously and were excluded from the analyses. For the ITS region, we used two different alignment strategies. Using SATé, we generated 100 alignments, of which the one yielding the tree with the best likelihood score (–18953.91) contained 1542 sites (ITS1∶1–800, 5.8S: 801–969, ITS2∶970–1542) and was retained, combined with nrLSU and EF1-alpha alignments and used in a Bayesian MCMC analysis. In the manual refinement strategy, we found that five iterations were sufficient to flat out changes in the alignments, which were reflected by alignment lengths, the topology of single-gene ML phylograms and Bayesian consensus trees. The guide tree had a profound effect on the inferred alignment length. In the first alignment step by PRANK, by using the first guide tree estimated from the data, the length of the resulting alignment contained 4478 sites, the second, computed with a guide tree estimated from the first alignment contained 3102 sites, whereas subsequent alignments, computed with guide trees estimated from all three genes were around 1500 bp long (the final being: ITS1∶1–730, 5.8S: 731–902, ITS2∶903–1614). To estimate the phylogenetic relationships in the Bolbitiaceae, this final ITS alignment has been concatenated with the two other loci. Alignments and phylogenetic trees have been uploaded to TreeBase (No: 13626).

We did not encounter significant conflict between the single-gene trees. To address whether the ITS region contains phylogenetic signal, or the alignment is merely a result of forcing unalignable sequences into an alignment, we inferred single-gene ITS trees with and without applying GBlocks as well as inferred trees from the recoded gap data only (Figures S1–S4). We found that these trees are congruent with each other (as judged by mutually exclusive clades with strong ML bootstrap support) as well as LSU and EF1-alpha trees, differing only in the number of strongly supported clades. This suggests that the ITS alignment contains phylogenetic signal, as opposed to the scenario where forced alignment would contain random noise. The final concatenated alignment thus contained all three genes, plus the recoded binary indel characters, totaling to 4075 nucleic acid sites and 864 presence/absence characters. Of the indel characters, 450 were parsimony informative. The nrLSU alignment of the agaricoid clade contained 1367 characters.

### Phylogenetic Analyses

For examining the phylogenetic distribution of coprinoid species, we downloaded all nrLSU sequences of the agaricoid clade from GenBank, excluding sequences shorter than 500 bp and those of environmental origin. The ML phylogeny completed within 24 hours, 425 and 252 branches receiving probabilities >0.90 and >0.95 from approximate likelihood ratio tests (aLRT). The phylogeny revealed many of the major agaricoid clades recovered by Matheny et al., [17], including the Inocybaceae, Psathyrellaceae, Cortinariaceae (paraphyletic), Bolbitiaceae, Crepidotaceae, Agaricaceae, Strophariaceae, *Hebeloma+Alnicola*, etc. Coprinoid lineages were found in the Agaricaceae, Bolbitiaceae and Psathyrellaceae.

**Figure 2:**
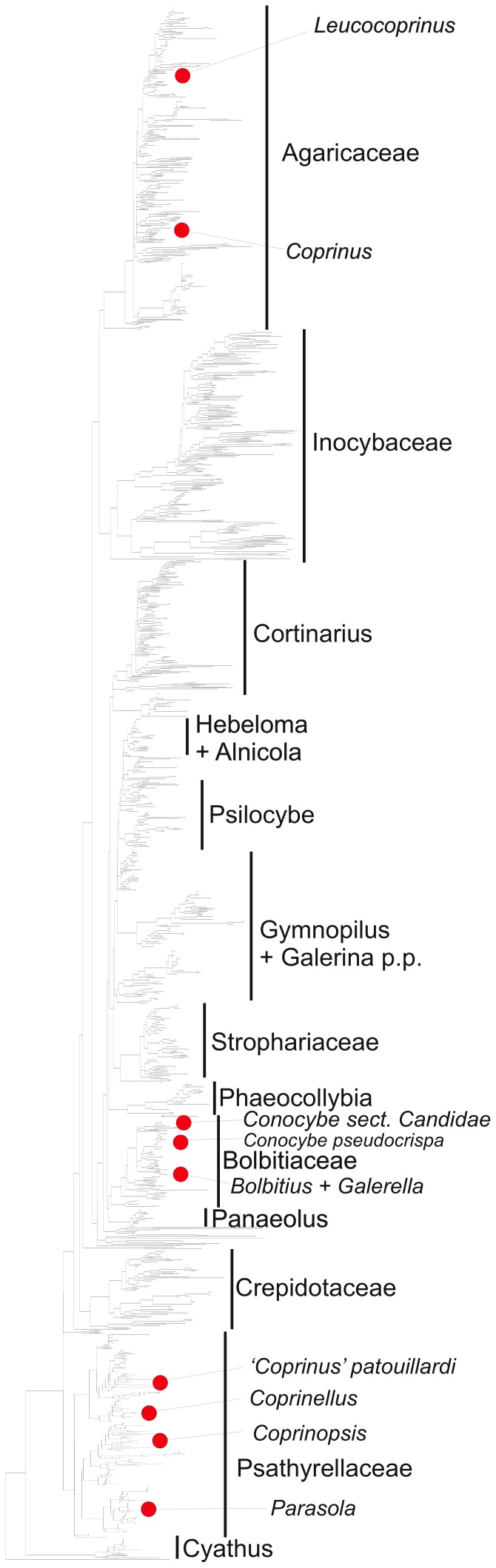
Phylogenetic distribution of the coprinoid fruiting body type in the agaricoid clade (sensu [**17**]) shown on a Maximum Likelihood tree inferred from LSU sequences of all taxa of the agaricoid clade present in Genbank, supplemented with LSU sequences generated in this and a previous study ([**26**]).

In total, we performed the following Bayesian runs for this study: five for each iteration of the manual alignment refinement, one for the SATé alignment, one for the gap data only, one for GBlocks-curated alignments as well as a BEAST analysis of the final nucleic acid dataset. All analyses converged sufficiently to the stationary distributions, so we established the burn-in as 15.000.000 generations. The consensus tree obtained in the final Bayesian analysis using MrBayes is presented in Fig. 2. All analyses provided strong support for both early and more recent nodes with a few exceptions. *Conocybe volviornata* had an ambiguous position, probably due to the amount of missing data for this species. In the following, BPP, BBPP and MLBS stand for Bayesian posterior probabilities inferred using MrBayes, Bayesian posterior probabilities inferred using BEAST and Maximum Likelihood bootstrap support inferred using RAxML for the final concatenated dataset. The analyses recovered eight major clades within the family, Pholiotina 1 (BPP:1.0, BBPP:1.0, MLBS:53%), Pholiotina 2 (BPP:1.0, BBPP:1.0, MLBS:100%), Pholiotina 3 (BPP: 0.94, BBPP:1.0, MLBS:61%), Bolbitius (BPP:1.0, BBPP:0.86, MLBS:100%), Conocybe 1 (BPP:1.0, BBPP:1.0, MLBS:71%), Conocybe 2 (BPP: 1.0, BBPP:1.0, MLBS:97%), Conocybe 3 (BPP:1.0, BBPP:1.0, MLBS: 83%), Conocybe 4 (BPP:0.95, BBPP:0.71, MLBS:90%), Conocybe 5 (BPP:1.0, BBPP:1.0, MLBS:100%) and Conocybe 6 (BPP:0.99, BBPP:1.0, MLBS: 64%). Of the three secotioid species (i.e. species with closed fruiting bodies, but well-developed lamellae) sequenced, *Conocybe deliquescens* ( = *Gastrocybe lateritia*) was nested in Conocybe clade 6, *C. cylindracea* in Conocybe clade 1, whereas *Galeropsis desertorum* clustered in the outgroup (Panaeoloidae). The two sequenced species of *Galerella* did not form a monophyletic clade of their own, *G. floriformis* appears basal within the family, whereas *G. nigeriensis* is close to the Pholiotina 2 clade.

Tree topologies of various analyses were largely congruent. The branching order around the Pholiotina 3 and *Bolbitius* plus Pholiotina 1 clades are, however somewhat uncertain, appearing in contradicting positions on the MrBayes and BEAST consensus trees, although the BPP-s were 1.0 for both alternatives. The placement of these clades on the Maximum Likelihood tree was congruent with the BEAST tree, but ML bootstrap support was lower than 50%.

The tree inferred from the recoded characters of indel presence/absence recovered the same major clades (Fig. S1), although the resolution of the 50% majority rule consensus tree was much lower (Fig. S2, which is well explained by the small number of characters (864, of which 450 was parsimony informative). This tree contained one large polytomy comprising the Conocybe 1–6 as well as the Pholiotina 3 clades. *Galerella floriformis* and the *Bolbitius*+Pholiotina 1 clades were supported as monophyletic, and appeared basal to the rest of the family. Since support was weak at these nodes, they do not imply significant conflict with the nucleotide-based analysis in statistical terms. Two specimens, lacking the ITS region were also placed in polytomy. Thus, we conclude that indel data are congruent with nucleic acids and provide a reliable signal with regard to the phylogeny of the Bolbitiaceae.

The tree inferred from the three-locus dataset with gapped sites deleted from the ITS region (by GBlocks) was congruent with trees inferred from the whole alignment (Figs. S3–S4).

### Constraint Analyses

We tested two morphologically informed hypotheses by constraint analyses. The first was the monophyly of the three volvate species *C. volvata, C. vaginata* and *C. volviornata*, which were inferred in different positions on the tree. Based on *p*-values of the approximately unbiased test, trees where these species appear as monophyletic cannot be rejected (*p* = 0.138–0.139). Since *C. volviornata* is the only species represented only by one locus, which is the most conserved of the three loci (nrLSU) we are inclined to attribute its ambiguous position to an effects of missing data.

The monophyly of *Pholiotina* could be rejected (*p-values*: 0.004–4*10^−5^). The constraint trees were designed to allow *Galerella nigeriensis* to be resolved outside *Pholiotina*, however, it was resolved within the latter genus. Similarly, forcing *Pholiotina* species without veil to be monophyletic results in significantly worse likelihoods (au test *p-values*: 0.001 for all 10 trees).

### Ancestral State Reconstruction

**Figure 3:**
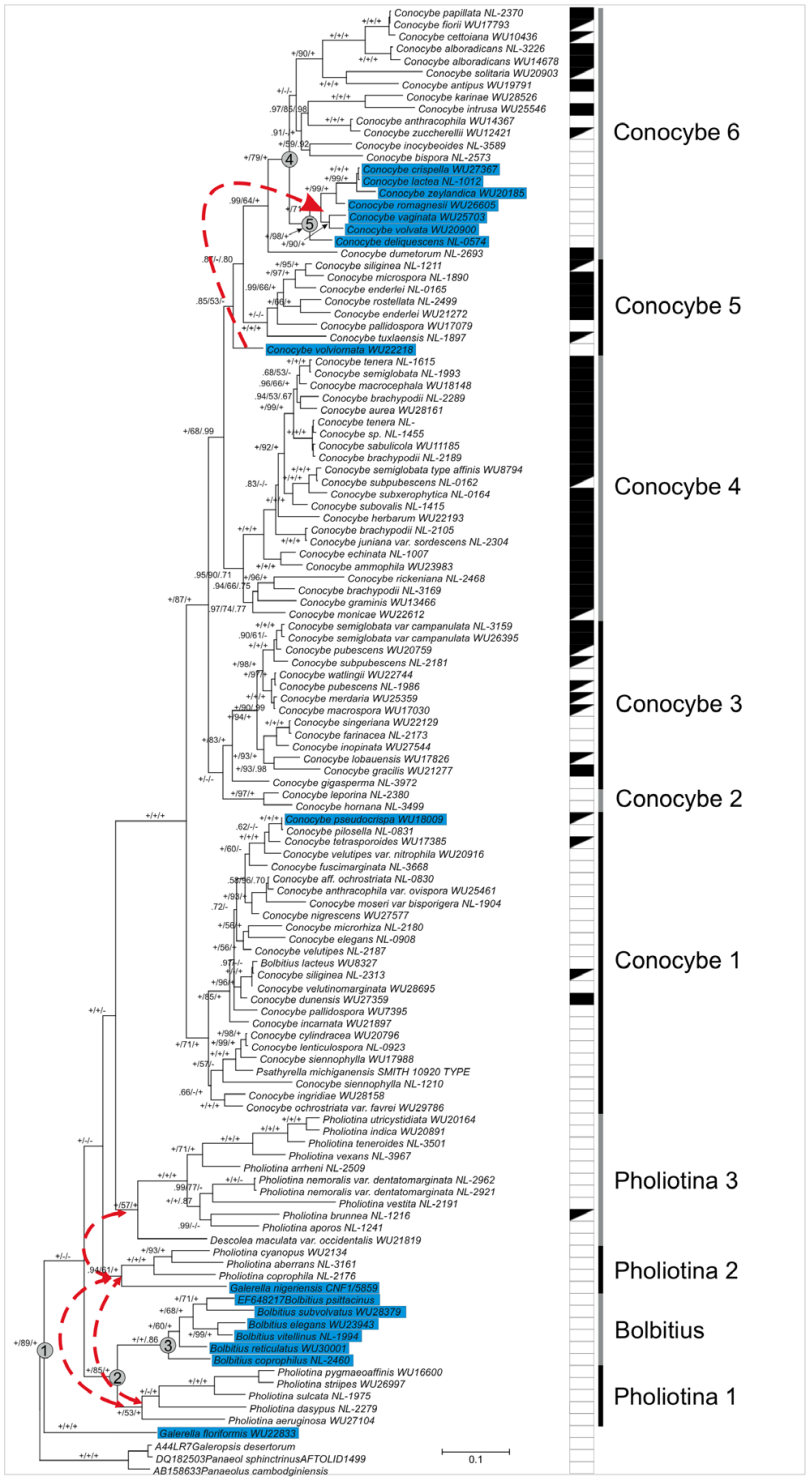
50% Majority rule consensus phylogram showing the phylogenetic relationships in the Bolbitiaceae. Branch support values are MrBayes posterior probabilities, ML bootstrap values, BEAST posterior probabilities, in order. Nodes for which ancestral fruiting body type has been reconstructed are labeled nodes 1–5, corresponding to the Bolbitiaceae, *Bolbitius*+*Pholiotina* 1, *Bolbitius*, *Conocybe* 6 and section *Candidae*. Taxa with coprinoid combination of characters (pseudoparaphyses, ephemeral fruiting bodies which collapse upon maturity, plicate cap surface) are highlighted in blue. Relationships tested by constraint analyses are marked by dashed lines. The composition of stipe covering either as hairs only (open squares), lecythiform cystidia only (filled squares) and both types (fountain fill) is shown to the right of the tree. Note that ML bootstrap values and Bayesian posterior probabilities from the BEAST runs were obtained without the indel data.

Ancestral states were reconstructed for five nodes on a sample of 1000 post-burn-in trees using ML, including the root node of the Bolbitiaceae (Node 1–5. Table 2, Fig. 3). Ancestral states and corresponding hypothesis tests are summarized in Table 2. The ancestral fruiting body type of the Bolbitiaceae was inferred as non-deliquescent with significant support (d(L_1_–L_2_) = 2.205), followed by independent emergence of the deliquescent physiology in the genus *Bolbitius*, section *Candidae* of *Conocybe*, *Bolbitius lacteus*, etc. Taken together, these reconstructions revealed six independent acquisitions of deliquescence in the Bolbitiaceae (Fig. 3). Given the uncertainties of the phylogeny, first of all, the ambiguous position of *C. volviornata* (see above) and *C. pseudocrispa*, however, this number may be biased upward. Consequently, the number of independent acquisitions of deliquescence drops to five when *C. volviornata* is monophyletic with *C. volvata* and *C. vaginata* and four when the placement of *C. pseudocrispa* (belonging to section *Candidae* based on morphology) is also regarded ambiguous.

The type of cheilocystidia was inferred as fusiform-utriform in all early nodes of the Bolbitiaceae, with one gain on the branch leading to *Conocybe* and one in the terminal branch of *Ph. brunnea*. Stipe covering was inferred to have undergone more changes with a mean of 11.3 and 14.2 state changes per tree under multistate and binary coding, respectively. Under binary coding, the number of gains/losses of lecythiform and hair-like cystidia was inferred as 5.29/2.18 and 0.40/5.76, respectively. Simultaneous loss of hairs and gain of lecythiform cystidia were inferred at a relatively low rate (mean 2.45 per tree) under multistate coding, which may explain the differences observed between the two coding regimes. The ancestral type of stipe coverage was estimated to having hairs only.

## Discussion

### Alignment Undermatching?

Progressive alignment methods are well-known to be sensitive to the order in which sequences are input to the calculation, most often determined by a guide tree which is estimated from the sequences to be aligned [35], [38], [57], [58]. Consistent with the expectations, our results show that the guide tree had a profound effect on the inferred alignment. However, hitherto documented instances of guide tree bias [40], [59] differ from what we have observed for PRANK. Unlike most other algorithms, PRANK do not overcondense the alignments by erroneously inferring spans of non-homologous sequences as homologous [34]. However, we observed that it infers false negative homologies, resulting in unrealistically long alignments. We term this phenomenon alignment undermatching, referring to alignment overmatching, a pattern observed for ClustalW and MAFFT [33]. The length of the alignment decreased when the accuracy of the guide tree increased (although we cannot assess accuracy objectively with the empirical data at hand, the trees inferred from a multigene alignment more likely to be correct than one based on genetic distances). The excessive length of the first alignments, including the one which PRANK produced after its built-in second iteration was caused by several, noticeably homologous blocks of sequences inferred as non-homologous, separated by gapped sites. These evidently biased alignment lengths upward and, although the proportion of false positive homologies is not expected to increase as a result of "alignment undermatching", the proportion of accurately aligned residues decrease, and thus the phylogenetic signal may be biased. Although PRANK is successful in avoiding nearby indels to collapse, which would result in overcondensed alignments (as is often encountered with ClustalW, MAFFT, etc.), the phenomenon called alignment overmatching by Löytynoja and Goldman [33] our results suggest that in the absence of a reasonably accurate guide tree, on the other hand, its strategy for distinguishing and fixing insertions and deletions can cause undermatching of the sequences, resulting in multiple homologous blocks inferred as separate insertion/deletion events. Because in PRANK, the distinction between insertions and deletions is made on the basis of an outgroup-rooted tree [33], [34], the accuracy of the guide tree becomes the primary determinant of how accurately indels are placed in the alignment. As our analyses predict, the guide trees calculated by PRANK may not always allow reasonably accurate gap placement.

Unambiguous parts of the alignment have recently been used to overcome this issue [40]. This is related to the approach proposed in this study, however, we used all data (including two easily alignable genes) to estimate new guide trees in an iterative framework. With regard to the results, our approach and another iterative alignment refinement strategy, SATé [31] performed similarly, converging to the same answer for the problem of ITS alignment. We conclude that improved guide trees are needed for alignment algorithms, and that even alignments produced by highly sophisticated algorithms, such as PRANK could be further improved by supplying a reasonably accurate guide tree or iterative refinement of a starting tree - alignment pair. This strategy allows the inference of improved alignments for datasets not tractable by using simultaneous phylogeny and alignment inference algorithms (such as Bali-Phy), which can more naturally handle this uncertainty in a Bayesian framework.

### Phylogenetic Relationships in the Bolbitiaceae

Our analyses included all but one genera (the exception being *Tubariella*) of the Bolbitiaceae. The analysis of >1500 nrLSU sequences of the agaricoid clade confirms the monophyly of the Bolbitiaceae in a restricted sense [11], that is, excluding *Agrocybe, Leratiomyces* and *Cyttarophyllum* [11], [12], [14], [16], [60]. These three genera show an affinity to *Hebeloma* and *Psilocybe* on the basis of the Maximum Likelihood tree (the sequence of *Agrocybe pediades* within *Stropharia* probably represents a misidentification). *Agrocybe* was not monophyletic on the ML tree. This is consistent with findings of Moncalvo et al. [14], Gulden et al. [13], Walther et al. [16] and provides clear evidence for the exclusion of *Agrocybe* from the Bolbitiaceae, as proposed by Walther and Weiss [11]. *Panaeolus, Panaeolina* and *Galeropsis* formed a sister group of the Bolbitiaceae.

Three analyzed species of *Descolea* (*D. gunnii, D. maculata, D. antarctica*), a genus classified either in the Cortinariaceae or the Bolbitiaceae, show an affinity to annulate *Pholiotina* species. This relationship is consistent with the macromorphology of the two clades, both are characterized by small and relatively slender fruiting bodies, with a bell-shaped pileus and a well-developed, grooved ring half-way on the stipe. On the other hand, the limoniform, ornamented spores and a putative mycorrhizal lifestyle [61], [62] represent shared features with the Cortinariaceae. Phylogenies seem to consistently support the placement of *Descolea* in the Bolbitiaceae [13], [14], [17], where it would represent the only ectomycorrhizal (ECM) lineage of the family.

The core Bolbitiaceae include the genera *Conocybe*, *Pholiotina*, *Bolbitius*, *Galerella*, *Descolea* as well as the sequestrate genus *Gastrocybe*. The latter has recently been recombined in *Conocybe* (as *C. deliquescens*, [5]), which was suggested by phylogenetic analyses of ITS sequences [3]. Its phylogenetic position is also verified by this study using three markers (ITS, 5′ LSU, EF1 alpha).

Out of the two species of *Galerella* included in this study, one was inferred as the basalmost clade in the Bolbitiaceae, while the other as a sister group of Pholiotina 2, implying polyphyly of its genus. The most important taxonomic character of *Galerella* is the brittle, ephemeral fruiting bodies with strikingly plicate - sulcate cap surface [4], [20], [63]. This suite of traits match that of coprinoid fruiting bodies completely, which has been shown to emerge convergently in several clades of the Agaricales [26], [28], [29]. In light of the convergent nature of coprinoid morphology, a straightforward interpretation of the phylogenetic relationships is that *Galerella*, as circumscribed now is an artificial grouping of species which convergently evolved the coprinoid morphology within various lineages of the Bolbitiaceae. This interpretation of the phylogenetic results is analogous to the case of *Coprinus* sensu lato [64], and should lead to the splitting of *Galerella*. However, more species and collections should be examined to confirm this result.

At genus level, *Pholiotina* seems to be a phylogenetically heterogeneous group, split into three clades on the Bayesian trees. Of these, Pholiotina 3 contains species with a rich veil, usually forming a spectacular ring on the stipe (sections *Vestitae*, *Pholiotina* and *Intermediae*, [4]). Pholiotina 1 and 2 contain species without veil, but instead with pileocystidia or a slimy cap surface (section *Cyanopodae*, *Piliferae*, *Vestitae* p.p., *Verrucisporae*). Representatives of these two clades are characterized by a poorly developed veil, which never forms a distinct annulus on the stipe. Constraint analyses forcing *Pholiotina* to be monophyletic returned low *p*-values (<<0.01), which underpins the need for splitting *Pholiotina* into smaller genera. In that case, the use of the generic name, originally given to an annulate taxon [65] should be restricted to species of Pholiotina 3 clade. The paraphyly of *Pholiotina* conclusively rejects classifications, which treat *Pholiotina* as a subgenus of *Conocybe* [7].

Species of *Bolbitius* form a well-supported clade (BPP: 1.0, BBPP: 0.86, MLBS: 100%) sister to Pholiotina 1. This finding is supported by the morphological uniformity of *Bolbitius*, i.e. viscid to glutinous pileus, which gets sulcate-plicate, the presence of pseudoparaphyses and a partial deliquescence or collapse of the mature fruiting bodies.

The genus *Conocybe* formed a large, well-supported clade (BPP: 1.0, BBPP: 1.0, MLBS: 100%). Based on the clade structure within *Conocybe*, we distinguish five subclades, however, the subclades do not correspond to the current infrageneric classification of the genus. The three major sections (*Conocybe, Pilosellae, Mixtae*) can be recognized in our trees, although none of them appears as monophyletic, suggesting that other morphological traits may serve better for circumscribing sections in *Conocybe*. Smaller sections defined on the basis of fragile, pale fruiting bodies (section *Candidae*) or the presence of volva (section *Singerella*) were inferred as monophyletic. The pattern seen in *Conocybe* raises the question how the composition of stipe covering evolved in the Bolbitiaceae. In addition to the stipe covering, the cystidia at the gill edge (cheilocystidia) are also of interest in this respect, since the same oil-flask-shaped cystidia characterize the genus *Conocybe*. Ancestral state reconstructions show that the hairs represent the ancestral cystidial morphology in the Bolbitiaceae, and lecythiform cystidia evolved multiple times. Lecythiform cheilocystidia show two gains, one in *Pholiotina brunnea*, the other in the most recent common ancestor of *Conocybe* species. Stipe covering shows a more complex evolutionary history. Both with additive binary and multistate coding, the lecythiform type of cystidia shows on average 5.2 gains and 2.1 losses per tree, whereas the hair-like morphology is lost more often than gained (mean gain/loss: 0.4/5.8). This, combined with evidence for the most recent common ancestor of *Conocybe* having had a stipe covering of hairs only, depicts an inverse relationship in the evolution of hair-like and lecythiform cystidia. That is, hair-like cystidia, the ancestral condition in *Conocybe* is gradually replaced by lecythiform cystidia. Although the difference between the adaptive value of these two character states is poorly understood, this evolutionary scenario suggests that the composition of stipe covering may have limited taxonomic value. Thus, we raise the possibility that for the infrageneric taxonomy of *Conocybe* other morphological traits should be sought, such as those used to delimit sections *Candidae* and *Singerella*, for which the phylogenetic analyses support monophyly. The presence of pseudoparaphyses in some species of section *Singerella* supports its monophyly with section *Candidae*. It is noteworthy that species of section *Singerella* have veil remnants at the stipe base (volva) or rarely on the margin of the cap. Interestingly, spurious veil remnants have been examined in *C. deliquescens* (section *Candidae*) also, which further supports the relatedness of the two groups.

It is noteworthy, that *C. dumetorum*, the single representative of section *Ochromarasmius* harbours a sister clade of Conocybe 6. This species is a representative of *Conocybe* species with rough to verrucose spores, often observable only under SEM [1], [4], [66].

### Deliquescent Lineages in the Agaricales

Deliquescence, i.e. the enzymatic autodigestion of mature fruiting bodies, is one of the several ways of departure from the usual pileate-stipitate fruiting body form in the Agaricales [26], [29], [67], [68]. Like gasteroid species [14], deliquescent lineages do not form a monophyletic group. Recently, a complex series of phenotypic changes have been identified which correlate with changes from non-deliquescent to deliquescent fruiting bodies, which include the evolution of pseudoparaphyses to replace basidioles, bimorphic basidia, accordion-like folded, plicate pileus surface and an increase in the size of hymenial cystidia [26]. The word coprinoidization have been proposed for the series of these changes following fruiting body evolution. All these changes are concordant in that they contribute to the acceleration of the ontogeny of the fungus by opening the way for rapid turgor manipulation in the inflating cells [25], [26]. Also, because of the apparent lack of a direct adaptive advantage imposed by autolysis itself, we hypothesized that it might be the rapid ontogeny that is an important aspect in the altered adaptation capabilities of deliquescent lineages as compared to non-deliquescent ones. Thus, we think coprinoid lineages should not only comprise those with a pronounced autodigestive phase, resulting in the well-known inky fluid, but also those in which the above mentioned syndrome of increase in hymenial cell-sizes and consequently a rapid cap expansion, can be observed. In support of this, partial deliquescence, or a sudden collapse of the fruiting body is often observable in species with no actual enzymatic decay but pseudoparaphyses, large hymenial cystidia, plicate cap surface and bimorphic basidia. Within the Psathyrellaceae, such taxa can be found in the genera *Parasola* and *Coprinellus* (e.g. *C. disseminatus)*. According to this logic, species of the genera *Bolbitius*, *Galerella* as well as section *Candidae* of *Conocybe* should be regarded as coprinoid. All of these taxa possess pseudoparaphyses, more or less enlarged hymenial cystidia and the surface of their cap is plicate, however bimorphic basidia are absent. In this study, the above mentioned species formed 4 clades, with strong support from both ML and Bayesian MCMC analyses. Thus, the present phylogeny extends the number of known coprinoid lineages with four in the Bolbitiaceae. Within the Psathyrellaceae, at least four deliquescent lineages have been identified, although uncertainty in the phylogeny raised the possibility of additional two lineages in *Coprinopsis* and *Coprinellus*. Within the Agaricaceae, *Coprinus* s. str. represents an additional deliquescent lineage, with 3–4 species, including the well-known *C. comatus*. An additional group of taxa with plicate cap surface, bi(tri-)morphic basidia and pseudoparaphyses is found in *Leucocoprinus* (Agaricaceae) [2], [69]. Molecular phylogenies suggested *Leucocoprinus* to have emerged from within *Leucoagaricus* ([14], [70], Fig. 2), from which it only differs in the presence of pseudoparaphyses, bimorphic basidia and the plicate cap surface. Both published phylogenies [14], [70] and the ML tree inferred in this study leave uncertainties about the monophyly of coprinoid *Leucocoprinus* species (i.e. *L. birnbaumii, L. brebissonii, L. fragilissimus*, not counting *Leucocoprinus spp* in the wide sense, for the nomenclatural controversies over this genus see [70]), and thus the number of coprinoid lineages they add to our understanding of the phylogenetic distribution of coprinoidization. Nevertheless, taken together, at least 10 independent lineages with coprinoid fruiting bodies have been identified in the Psathyrellaceae (∼4), Bolbitiaceae (∼4) and in the Agaricaceae (∼2). Interestingly, all these lineages appear among brown-spored mushrooms in the agaricoid clade (sensu [17]). The occurrence of multiple distantly related lineages that convergently acquired the same mechanism of adaptation suggests the presence of easily accessible pathway(s) of developmental specialization. Whether coprinoidization and the accelerated ontogeny are achieved by the same cellular or subcellular mechanisms in the various lineages is a question with a potential to reveal some of the general mechanisms of fruiting body evolution, but requires more research on the ontogeny and subcellular mechanisms of the species involved.

## Supporting Information

Figure S1 **50% Majority Rule phylogram inferred with gapped sites of the ITS alignment AND recoded gap characters excluded from the analysis (in MrBayes).**
(DOCX)

Figure S2 **50% Majority Rule phylogram inferred from recoded binary gap characters of the ITS alignment. A total of 864 characters (450 parsimony informative) of gap presence/absence (0/1) were used for the analysis (in MrBayes).**
(DOCX)

Figure S3 **Maximum Likelihood Phylogram inferred from the concatenated three-locus dataset, without the indel characters (from RAxML).**
(DOCX)

Figure S4 **Maximum Clade Credibility tree from the BEAST analysis of the concatenated three-locus dataset. Note that branch lengths were omitted from the figure.**
(DOCX)

File S1 **List of Accession Numbers of GenBank sequences used for assessing the phylogenetic distribution of coprinoid fruiting bodies.**
(DOCX)
