# Supplementary material for: Iteratively Refined Guide Trees Help Improving Alignment and Phylogenetic Inference in the Mushroom Family Bolbitiaceae
Source: PLoS One. 2013 Feb 13;8(2):e56143. doi: 10.1371/journal.pone.0056143 (PMC3572013; doi:10.1371/journal.pone.0056143)
Supplement: Figure S1 — 50% Majority Rule phylogram inferred with gapped sites of the ITS alignment AND recoded gap characters excluded from the analysis (in MrBayes). (DOCX) [file pone.0056143.s001.docx]

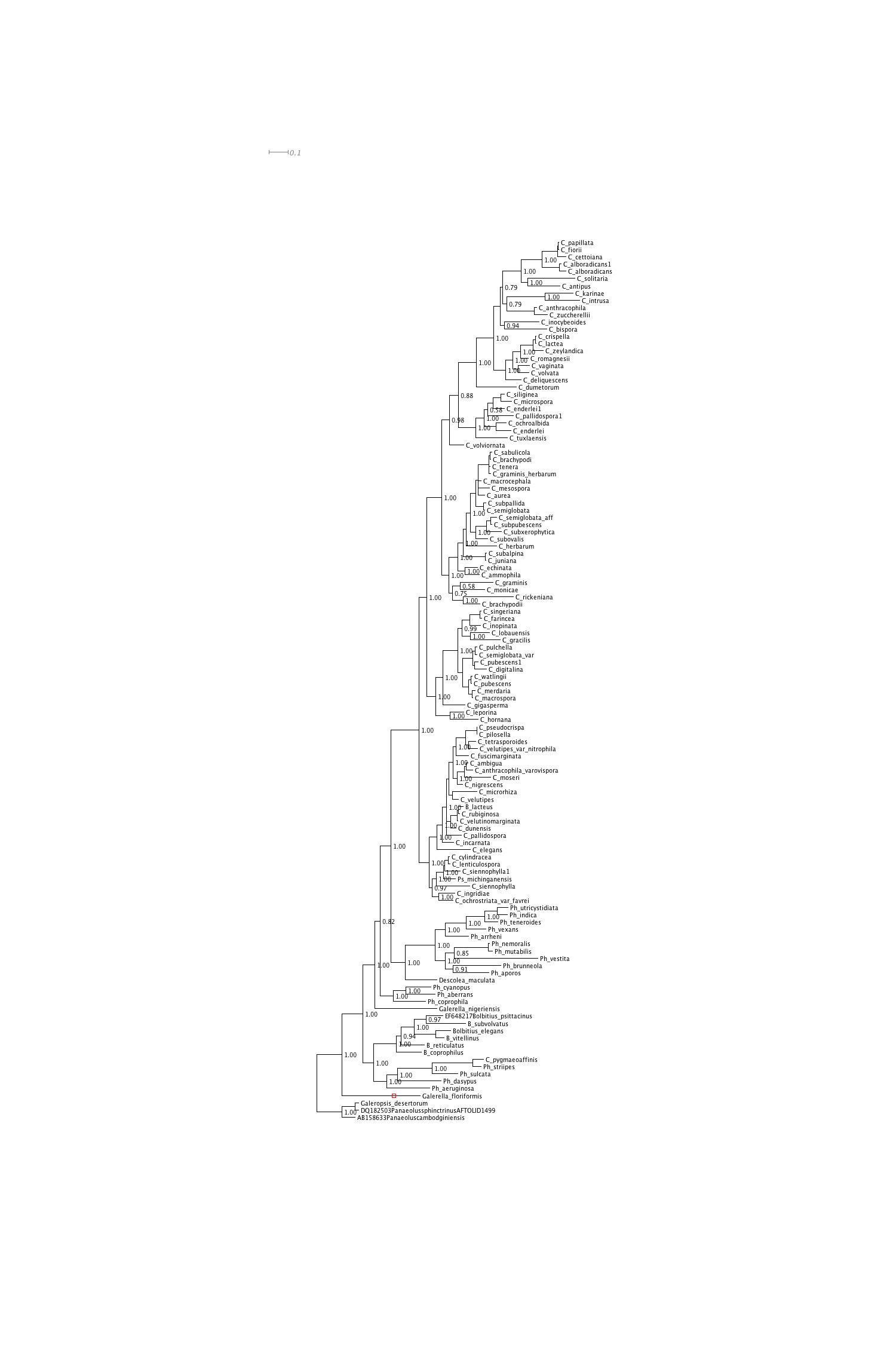


Figure S1. 50% Majority Rule phylogram inferred with gapped sites of the ITS alignment AND recoded gap characters excluded from the analysis (in MrBayes).
