# Supplementary material for: Iteratively Refined Guide Trees Help Improving Alignment and Phylogenetic Inference in the Mushroom Family Bolbitiaceae
Source: PLoS One. 2013 Feb 13;8(2):e56143. doi: 10.1371/journal.pone.0056143 (PMC3572013; doi:10.1371/journal.pone.0056143)
Supplement: Figure S2 — 50% Majority Rule phylogram inferred from recoded binary gap characters of the ITS alignment. A total of 864 characters (450 parsimony informative) of gap presence/absence (0/1) were used for the analysis (in MrBayes). (DOCX) [file pone.0056143.s002.docx]

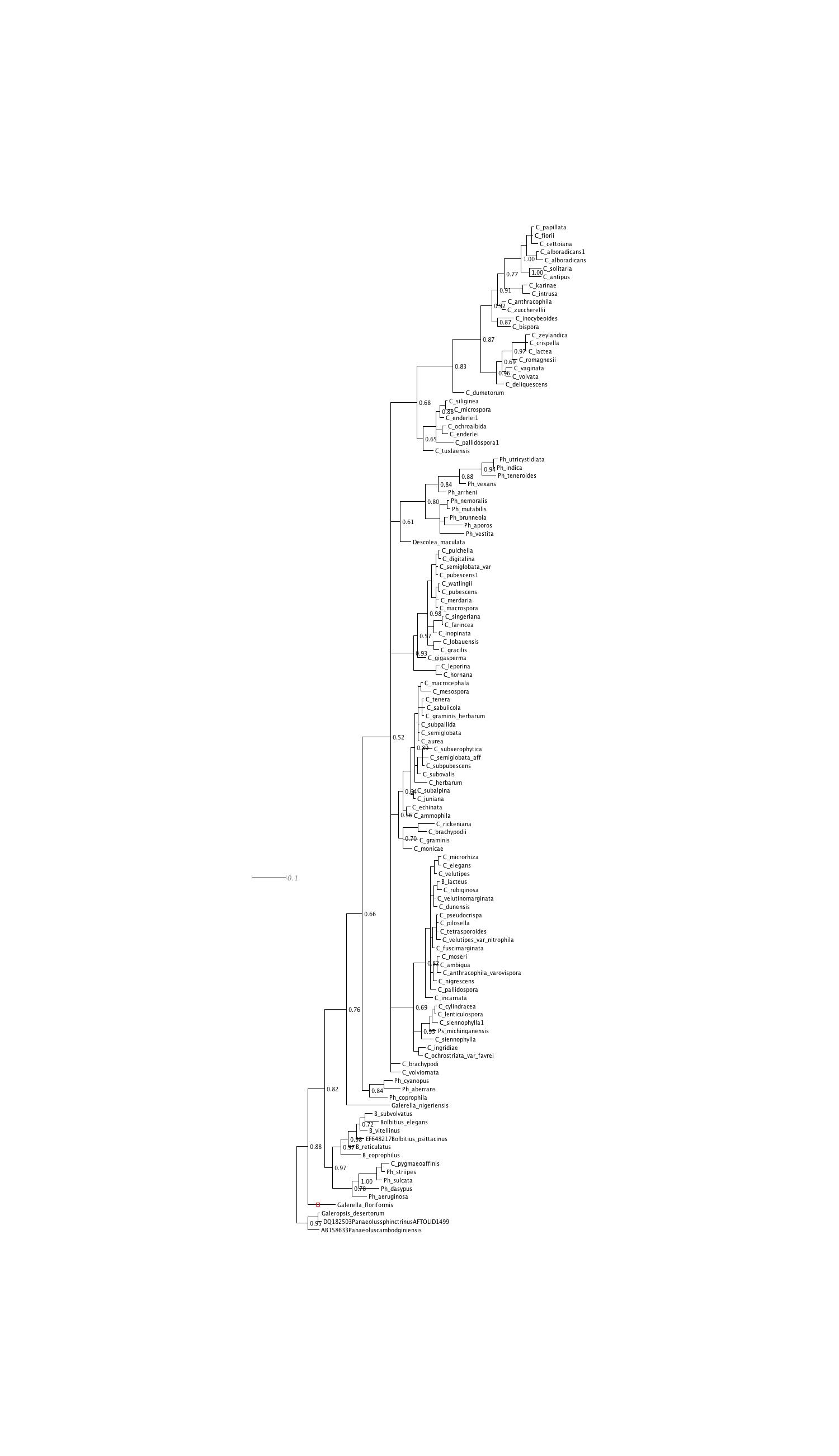


Figure S2. 50% Majority Rule phylogram inferred from recoded binary gap characters of the ITS alignment. A total of 864 characters (450 parsimony informative) of gap presence/absence (0/1) were used for the analysis (in MrBayes).
