# Supplementary material for: Iteratively Refined Guide Trees Help Improving Alignment and Phylogenetic Inference in the Mushroom Family Bolbitiaceae
Source: PLoS One. 2013 Feb 13;8(2):e56143. doi: 10.1371/journal.pone.0056143 (PMC3572013; doi:10.1371/journal.pone.0056143)
Supplement: Figure S3 — Maximum Likelihood Phylogram inferred from the concatenated three-locus dataset, without the indel characters (from RAxML). (DOCX) [file pone.0056143.s003.docx]

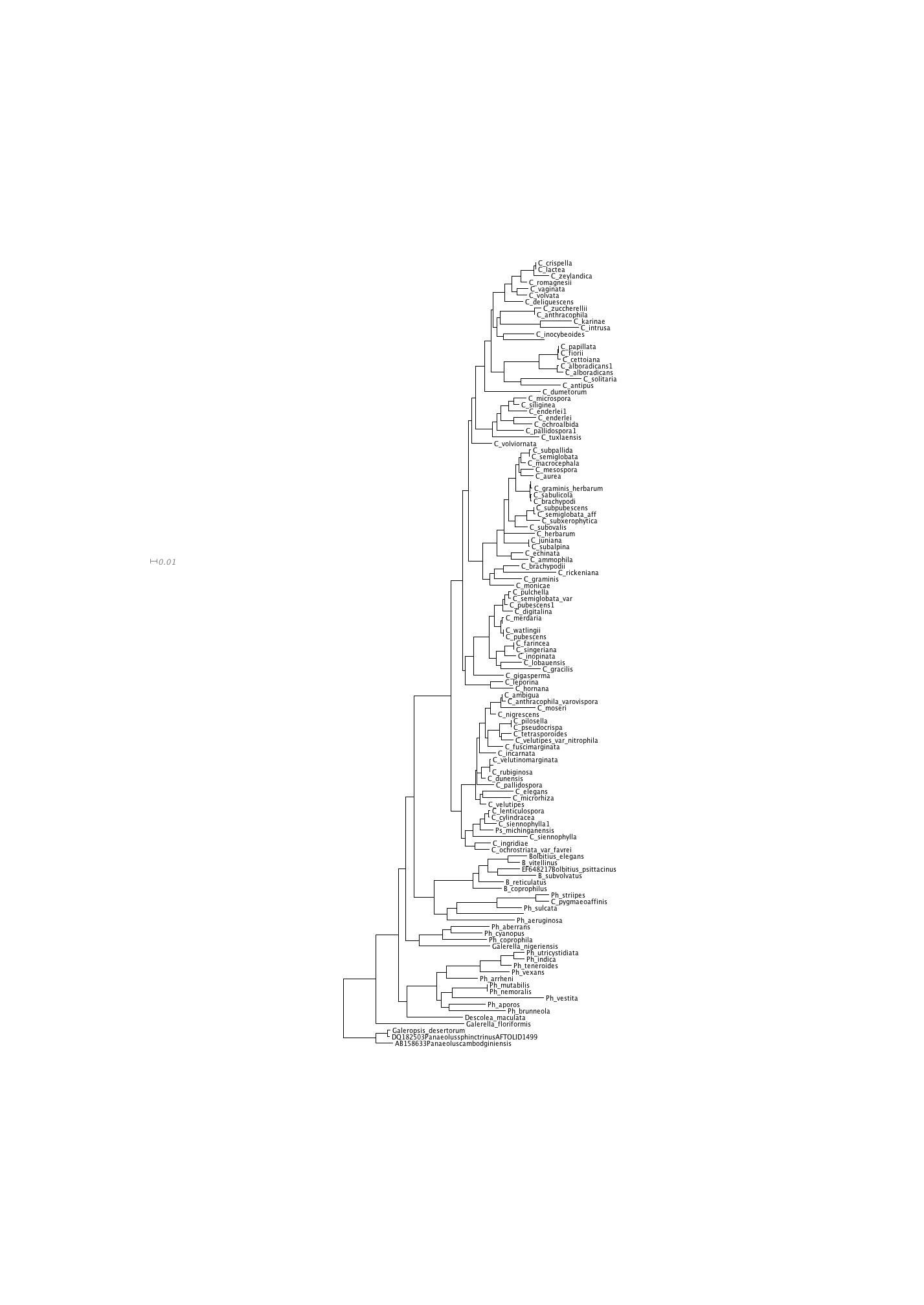


Figure S3. Maximum Likelihood Phylogram inferred from the concatenated three-locus dataset, without the indel characters (from RAxML)
