# Supplementary material for: Iteratively Refined Guide Trees Help Improving Alignment and Phylogenetic Inference in the Mushroom Family Bolbitiaceae
Source: PLoS One. 2013 Feb 13;8(2):e56143. doi: 10.1371/journal.pone.0056143 (PMC3572013; doi:10.1371/journal.pone.0056143)
Supplement: Figure S4 — Maximum Clade Credibility tree from the BEAST analysis of the concatenated three-locus dataset. Note that branch lengths were omitted from the figure. (DOCX) [file pone.0056143.s004.docx]

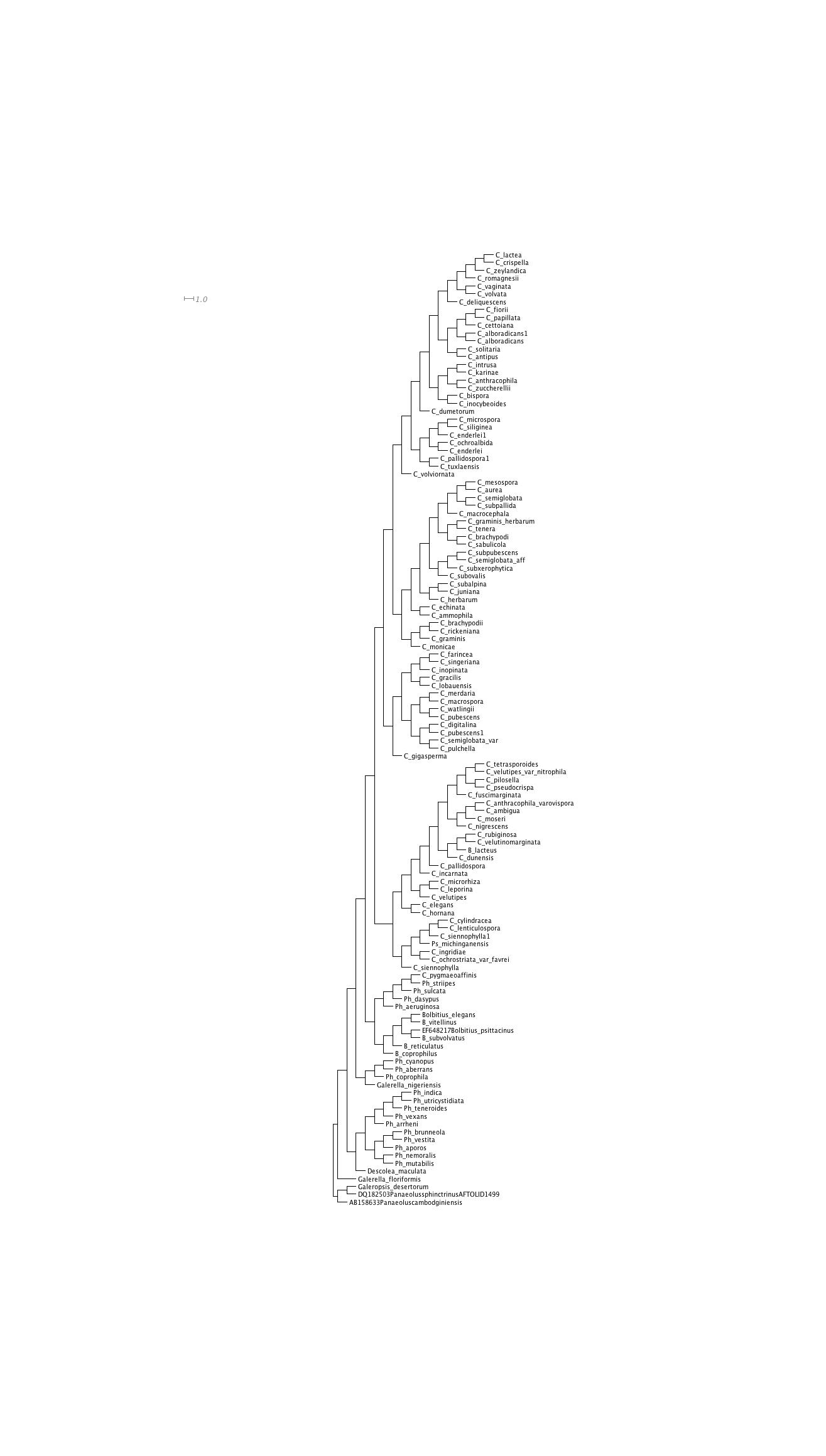


Figure S4. Maximum Clade Credibility tree from the BEAST analysis of the concatenated three-locus dataset. Note that branch lengths were omitted from the figure.
