# Supplementary material for: Iteratively Refined Guide Trees Help Improving Alignment and Phylogenetic Inference in the Mushroom Family Bolbitiaceae
Source: PLoS One. 2013 Feb 13;8(2):e56143. doi: 10.1371/journal.pone.0056143 (PMC3572013; doi:10.1371/journal.pone.0056143)
Supplement: File S1 — List of Accession Numbers of GenBank sequences used for assessing the phylogenetic distribution of coprinoid fruiting bodies. (DOCX) [file pone.0056143.s005.docx]

**List of Accession Numbers of GenBank sequences used for assessing the phylogenetic distribution of coprinoid fruiting bodies:**

AB073535 AB104646 AB104647 AB104648 U11917 AB104649 AB104650 AB104651 AB104734 ABU11911 AF041484 AF041485 AF041486 AF041487 AF041488 AF041489 AF041490 AF041491 AF041492 AF041493 AF041494 AF041495 AF041496 AF041497 AF041498 AF041499 AF041500 AF041501 AF041502 AF041503 AF041504 AF041505 AF041506 AF041507 AF041508 AF041509 AF041510 AF041511 AF041512 AF041513 AF041514 AF041515 AF041516 AF041517 AF041518 AF041519 AF041520 AF041521 AF041522 AF041523 AF041524 AF041525 AF041526 AF041527 AF041528 AF041529 AF041529 AF041530 AF041530 AF041531 AF041532 AF041533 AF041534 AF041535 AF041536 AF041537 AF041538 AF041539 AF041539 AF041540 AF041540 AF041541 AF041541 AF041542 AF041542 AF041544 AF041544 AF041545 AF041546 AF042009 AF042011 AF042013 AF042567 AF042568 AF042569 AF042570 AF042600 AF042613 AF042613 AF042614 AF042615 AF042616 AF042617 AF042618 AF042619 AF042627 AF042644 AF042644 AF056456 AF056457 AF056458 AF056458 AF056459 AF056459 AF056460 AF056460 AF059231 AF059231 AF079591 AF079591 AF079592 AF079592 AF079593 AF079593 AF079594 AF079594 AF079595 AF079595 AF079596 AF079596 AF079597 AF079597 AF079598 AF079598 AF079599 AF079599 AF079600 AF079600 AF079601 AF079601 AF079602 AF079602 AF079603 AF079603 AF079604 AF079604 AF079605 AF079605 AF079606 AF079606 AF079607 AF079607 AF079608 AF079608 AF079609 AF079609 AF079610 AF079610 AF079611 AF079611 AF079612 AF079612 AF079613 AF079613 AF079614 AF079614 AF079615 AF079615 AF079616 AF079616 AF079617 AF079617 AF079618 AF079618 AF079619 AF079619 AF079620 AF079620 AF079621 AF079621 AF079622 AF079622 AF079623 AF079623 AF079624 AF079624 AF079625 AF079625 AF079626 AF079626 AF079627 AF079627 AF079628 AF079628 AF079629 AF079629 AF079630 AF079630 AF079631 AF079631 AF079632 AF079632 AF079633 AF079633 AF079634 AF079634 AF079635 AF079635 AF079636 AF079636 AF079637 AF079637 AF079638 AF079638 AF079639 AF079639 AF079640 AF079640 AF079641 AF079641 AF079642 AF079642 AF079643 AF079643 AF079644 AF079644 AF079645 AF079645 AF079646 AF079646 AF079647 AF079647 AF079648 AF079648 AF079649 AF079649 AF079650 AF079650 AF079651 AF079651 AF079652 AF079652 AF079653 AF079653 AF079654 AF079654 AF079655 AF079655 AF079656 AF079656 AF079657 AF079657 AF079658 AF079658 AF079659 AF079659 AF079660 AF079660 AF079661 AF079661 AF079662 AF079662 AF079663 AF079663 AF079664 AF079664 AF079665 AF079665 AF079666 AF079666 AF079667 AF079667 AF079668 AF079668 AF079669 AF079669 AF079670 AF079670 AF079671 AF079671 AF079672 AF079672 AF139941 AF139941 AF139942 AF139944 AF139944 AF139946 AF139946 AF139972 AF139972 AF139976 AF139976 AF205669 AF205670 AF205671 AF205672 AF205673 AF205674 AF205675 AF205676 AF205677 AF205678 AF205679 AF205680 AF205681 AF205682 AF205683 AF205684 AF205685 AF205686 AF205687 AF205688 AF205689 AF205690 AF205691 AF205692 AF205693 AF205694 AF205695 AF205696 AF205697 AF205698 AF205699 AF205700 AF205701 AF205702 AF205703 AF205704 AF205706 AF205707 AF205708 AF205709 AF205710 AF205711 AF205712 AF208533 AF208534 AF261289 AF261289 AF261308 AF261308 AF261380 AF261381 AF261408 AF261431 AF261473 AF261474 AF261476 AF261477 AF261478 AF261479 AF261480 AF261481 AF261482 AF261483 AF261484 AF261485 AF261486 AF261487 AF261488 AF261489 AF261490 AF261491 AF261492 AF261493 AF261496 AF261497 AF261498 AF261499 AF261500 AF261501 AF261502 AF261503 AF261504 AF261505 AF261506 AF261508 AF261509 AF261510 AF261511 AF261512 AF261513 AF261514 AF261515 AF261516 AF261517 AF261518 AF261519 AF261520 AF261521 AF261521 AF261522 AF261523 AF261524 AF261525 AF261526 AF261527 AF261528 AF261549 AF261550 AF261551 AF261552 AF261582 AF261583 AF261594 AF261595 AF261596 AF261597 AF261598 AF261599 AF261600 AF261601 AF261602 AF261603 AF261604 AF261605 AF261606 AF261607 AF261608 AF261609 AF261610 AF261611 AF261612 AF261613 AF261614 AF261615 AF261616 AF261617 AF261618 AF261619 AF261620 AF261621 AF261622 AF261623 AF261624 AF261625 AF261626 AF261627 AF261628 AF261629 AF261630 AF261631 AF261632 AF261633 AF261634 AF261635 AF261636 AF261637 AF261638 AF261639 AF261640 AF261641 AF261642 AF261643 AF261644 AF261645 AF261646 AF261647 AF261648 AF261649 AF261650 AF261651 AF261652 AF261653 AF287873 AF291286 AF291287 AF291306 AF291307 AF291342 AF291344 AF291368 AF336246 AF336247 AF336255 AF336256 AF336257 AF336258 AF336272 AF352047 AF367931 AF367932 AF367934 AF367935 AF367936 AF367937 AF367938 AF367939 AF367940 AF367941 AF367942 AF367943 AF367944 AF367946 AF367947 AF367948 AF367949 AF367950 AF367952 AF367953 AF367954 AF367955 AF367956 AF367957 AF367958 AF367959 AF367960 AF367961 AF367962 AF367963 AF388742 AF388743 AF388744 AF388745 AF388746 AF388747 AF388748 AF388749 AF388750 AF388751 AF388752 AF388753 AF388754 AF388755 AF388756 AF388757 AF388758 AF388759 AF388760 AF388761 AF388762 AF388763 AF388764 AF388765 AF388766 AF388767 AF388768 AF388769 AF388770 AF388771 AF388772 AF388773 AF388774 AF388775 AF388776 AF388777 AF388778 AF388779 AF388780 AF388781 AF388782 AF388783 AF388784 AF388785 AF388786 AF407164 AF430268 AF430275 AF430281 AF430282 AF482876 AF482877 AF482878 AF482879 AF482880 AF482881 AF482882 AF482883 AF482884 AF482885 AF482886 AF482887 AF482888 AF482889 AF482890 AF482891 AF482892 AF482893 AF482894 AF482895 AF482896 AF518603 AF518622 AF518628 AF518630 AF518654 AF518663 U85273 AJ242644 AJ244517 AJ244518 AJ244519 AJ244520 AJ244521 AJ244522 AJ244523 AJ244606 AJ244607 AJ244608 AJ244609 AJ244611 AJ244612 AJ244613 AJ244614 AJ244615 AJ244616 AJ244617 AJ244618 AJ244619 AJ244620 AJ244621 AJ244622 AJ244623 AJ244624 AJ244625 AJ406551 AJ406565 AJ406566 AJ406583 AJ406584 AJ406585 AJ406588 AJ534929 AJ871493 AJ871494 AJ871495 AJ871496 AJ871497 AJ871498 AJ871499 AJ871500 AJ871501 AJ871502 AJ871503 AJ871504 AJ871505 AJ871506 AJ871507 AJ871508 AJ871509 AJ871510 AJ871511 AJ871512 AJ871513 AJ871514 AJ871515 AJ871516 AJ871517 AJ871518 AJ871519 AJ871520 AJ871521 AJ871522 AJ871523 AJ871524 AJ871525 AJ871526 AJ871527 AJ871528 AJ871529 AJ871530 AJ871531 AJ871532 AJ871533 AJ871534 AJ871535 AJ871536 AJ871537 AJ871538 AJ871539 AJ871540 AJ871541 AJ871542 AJ871543 AJ871544 AJ871545 AJ871546 AJ871547 AJ871548 AJ871549 AJ871550 AJ871551 AJ871552 AJ871553 AJ871554 AJ871555 AJ871556 AJ871557 AJ871558 AJ871559 AJ871560 AJ871561 AJ871562 AJ871563 AJ871564 AJ871565 AJ871566 AJ871567 AJ871568 AJ871569 AJ871570 AJ871571 AM747624 AM946419 AM946421 AM946422 AM946423 AM946424 AM946425 AM946426 AM946427 AM946428 AM946429 AM946430 AM946431 AM946432 AM946433 AM946434 AM946435 AM946436 AM946437 AM946438 AM946440 AM946441 AM946442 AM946443 AM946444 AM946445 AM946447 AM946454 AM946456 AM946459 AM946464 AM946465 AM946476 AY004233 AY029706 AY029707 AY033126 AY033127 AY033128 AY033129 AY033130 AY033131 AY033133 AY033134 AY033135 AY033136 AY033137 AY033138 AY033139 AY038309 AY038310 AY038312 AY038313 AY038315 AY038316 AY038317 AY038319 AY038321 AY038323 AY038324 AY038325 AY038326 AY038327 AY038328 AY038329 AY127749 AY127750 AY145853 AY145854 AY176342 AY176344 AY176345 AY176348 AY176349 AY176353 AY176356 AY176358 AY176360 AY176362 AY176364 AY176365 AY176367 AY176369 AY176371 AY176373 AY176376 AY176378 AY176380 AY176381 AY176383 AY176385 AY176387 AY176389 AY176391 AY176393 AY176395 AY176396 AY176398 AY176399 AY176401 AY176403 AY176404 AY176406 AY176409 AY176411 AY176412 AY176414 AY176416 AY176418 AY176421 AY176423 AY176425 AY176427 AY176429 AY176431 AY176433 AY176435 AY176437 AY176441 AY176443 AY176445 AY176446 AY176448 AY176450 AY176455 AY176456 AY176457 AY207134 AY207135 AY207136 AY207137 AY207138 AY207139 AY207140 AY207141 AY207142 AY207143 AY207148 AY207169 AY207169 AY207170 AY207170 AY207171 AY207171 AY207172 AY207172 AY207173 AY207173 AY207174 AY207174 AY207175 AY207175 AY207176 AY207176 AY207177 AY207177 AY207178 AY207178 AY207179 AY207180 AY207181 AY207182 AY207183 AY207184 AY207185 AY207186 AY207187 AY207188 AY207189 AY207190 AY207191 AY207192 AY207193 AY207195 AY207196 AY207201 AY207202 AY207203 AY207204 AY207205 AY207206 AY207207 AY207208 AY207211 AY207212 AY207213 AY207214 AY207215 AY207216 AY207217 AY207218 AY207219 AY207220 AY207226 AY207227 AY207230 AY207231 AY207232 AY207233 AY207263 AY207264 AY207265 AY207266 AY207268 AY207269 AY207270 AY207271 AY207272 AY207273 AY207274 AY207275 AY207277 AY207279 AY207280 AY207281 AY207282 AY207283 AY207284 AY207285 AY207286 AY207287 AY207288 AY207289 AY207290 AY207291 AY207292 AY207293 AY207294 AY207295 AY207298 AY207300 AY207301 AY207302 AY207303 AY207310 AY207311 AY219581 AY219582 AY219583 AY219584 AY219585 AY219586 AY219587 AY219588 AY219589 AY219590 AY219591 AY219592 AY219593 AY219594 AY219595 AY219596 AY219597 AY219598 AY219599 AY219600 AY219601 AY219602 AY219603 AY219604 AY219605 AY219606 AY219607 AY219608 AY219609 AY219610 AY219611 AY219612 AY219613 AY219614 AY219615 AY222740 AY239018 AY239019 AY239020 AY239021 AY239022 AY239023 AY239024 AY239025 AY239026 AY239027 AY293173 AY293174 AY293616 AY372212 AY372213 AY372214 AY372215 AY372216 AY380360 AY380361 AY380362 AY380363 AY380364 AY380365 AY380366 AY380367 AY380368 AY380369 AY380370 AY380371 AY380372 AY380373 AY380374 AY380375 AY380377 AY380378 AY380379 AY380380 AY380381 AY380382 AY380383 AY380384 AY380385 AY380386 AY380387 AY380388 AY380389 AY380390 AY380391 AY380392 AY380394 AY380395 AY380396 AY380397 AY380398 AY380399 AY380400 AY380401 AY380402 AY380403 AY380404 AY380405 AY380405 AY380406 AY380408 AY380409 AY380409 AY380410 AY388643 AY509115 AY509119 AY536280 AY571018 AY571019 AY575918 AY575919 AY586680 AY586681 AY586689 AY586690 AY586697 AY586697 AY586698 AY635763 AY635764 AY635765 AY635766 AY635772 AY635774 AY635774 AY635775 AY645058 AY646101 AY646101 AY646102 AY663836 AY663837 AY663838 AY668957 AY684151 AY684152 AY700186 AY700187 AY700190 AY700196 AY700198 AY702013 AY702014 AY702015 AY727536 AY732208 AY732209 AY732210 AY732211 AY732212 AY732213 AY745700 AY745703 AY745706 AY745707 AY748868 AY748869 AY750168 AY751566 AY818353 AY820890 AY833409 AY900113 DQ006270 DQ071685 DQ071685.2 DQ071686 DQ071687 DQ071688 DQ071689 DQ071690 DQ071692.2 DQ071693 DQ071693 DQ071694 DQ071695 DQ071696.2 DQ071699 DQ071700 DQ071704 DQ071706 DQ071709 DQ071711 DQ071740 DQ071740 DQ094787 DQ110871 DQ110872 DQ110872 DQ110873 DQ110873 DQ110874 DQ133941 DQ154108 DQ156128 DQ177285 DQ273436 DQ273437 DQ273507 DQ273508 DQ273509 DQ273510 DQ273511 DQ273512 DQ273513 DQ273514 DQ273515 DQ327649 DQ389731 DQ411537 DQ457653 DQ457660 DQ457660 DQ457660 DQ457661 DQ457662 DQ457664 DQ457666 DQ457666 DQ457668 DQ457668 DQ457669 DQ457680 DQ457681 DQ457681 DQ457684 DQ457685 DQ463323 DQ463324 DQ463325 DQ463326 DQ463327 DQ463328 DQ463329 DQ463330 DQ463331 DQ463332 DQ463333 DQ463334 DQ463335 DQ463336 DQ463337 DQ463338 DQ463339 DQ470815 DQ470817 DQ470817 DQ470818 DQ470818 DQ470819 DQ470822 DQ470823 DQ470825 DQ536415 DQ674802 DQ674808 DQ911597 DQ911601 DQ986225 DQ986226 DQ986227 DQ986228 DQ986229 DQ986230 DQ986231 DQ986232 DQ986233 DQ986234 DQ986235 DQ986236 DQ986237 DQ986238 DQ986239 DQ986240 DQ986241 DQ986242 DQ986243 DQ986244 DQ986245 DQ986246 DQ986247 DQ986248 DQ986249 DQ986250 DQ986251 DQ986252 DQ986253 DQ986254 DQ986255 DQ986256 DQ986257 DQ986258 DQ986259 DQ986260 DQ986261 DQ986262 DQ986263 DQ986264 DQ986265 DQ986266 DQ986267 DQ986268 DQ986269 DQ986270 DQ986271 DQ986272 DQ986273 DQ986274 DQ986293 DQ986294 DQ986294 DQ986295 DQ986296 DQ986297 DQ986298 DQ986299 DQ986300 DQ986301 DQ986302 DQ986303 DQ987902 DQ987903 DQ987904 DQ987905 DQ987906 EF051051 EF051052 EF051053 EF051054 EF051055 EF080873 EF532404 EF532405 EF535265 EF535272 EF535272 EF535276 EF535279 EF537889 EF561631 EF561632 EF561633 EF561634 EF613554 EF648217 EF648218 EU019232 EU019233 EU019234 EU019235 EU029949 EU183546 EU284015 EU284016 EU307813 EU307814 EU307818 EU307819 EU307822 EU307824 EU307825 EU307826 EU307827 EU307828 EU307831 EU307834 EU307835 EU307837 EU307838 EU307840 EU307841 EU307845 EU307848 EU307851 EU339310 EU433887 EU499601 EU499602 EU499603 EU499604 EU499605 EU499606 EU499607 EU499608 EU499609 EU499610 EU499611 EU499612 EU499613 EU499614 EU499615 EU499616 EU499617 EU499618 EU522726 EU522732 EU522733 EU522736 EU522741 EU522742 EU522744 EU522747 EU522749 EU522753 EU522754 EU522755 EU522759 EU522762 EU522764 EU522765 EU522766 EU522772 EU522774 EU522779 EU522782 EU522787 EU522791 EU522796 EU522804 EU522805 EU522819 EU522828 EU522829 EU522830 EU522833 EU522837 EU522842 EU522843 EU522845 EU522846 EU522848 EU555442 EU555443 EU555444 EU555446 EU555447 EU555448 EU555449 EU555452 EU555454 EU555456 EU555457 EU555458 EU555461 EU555463 EU555466 EU555468 EU555469 EU555471 EU555474 EU569834 EU569835 EU569836 EU569838 EU569840 EU569843 EU569845 EU569846 EU569847 EU569850 EU569853 EU569854 EU569855 EU569857 EU569858 EU569860 EU569868 EU569870 EU569871 EU569874 EU569875 EU600834 EU600837 EU600840 EU600842 EU600844 EU600851 EU600852 EU600853 EU600854 EU600856 EU600859 EU600861 EU600863 EU600865 EU600868 EU600870 EU600874 EU600877 EU600879 EU600880 EU600883 EU600884 EU600885 EU600890 EU600894 EU600895 EU600897 EU600903 EU604546 EU652377 EU652378 EU652393 EU652394 EU652396 EU652397 EU652400 EU664994 EU669269 EU669270 EU669271 EU669272 EU669282 EU669283 EU669284 EU669285 EU669286 EU669287 EU669288 EU669289 EU669290 EU669291 EU669292 EU669395 EU669396 EU669397 EU669398 EU669399 EU669400 EU669401 EU669402 EU669403 EU669404 EU669405 EU669406 EU669407 EU669409 EU669410 EU669411 EU681185 EU681188 EU712176 EU712281 EU712419 EU712474 EU712499 EU712517 EU712539 EU712573 EU712591 EU712598 EU727143 EU834287 EU846318 EU85278 FJ436322 FJ436324 FM160680 FM160681 FM160682 FM160683 FM160684 FM160685 FM160686 FM160687 FM160688 FM160689 FM160690 FM160691 FM160692 FM160693 FM160694 FM160695 FM160696 FM160697 FM160698 FM160699 FM160700 FM160701 FM160702 FM160703 FM160704 FM160705 FM160706 FM160707 FM160708 FM160709 FM160710 FM160711 FM160712 FM160713 FM160714 FM160715 FM160716 FM160717 FM160719 FM160720 FM160721 FM160722 FM160723 FM160724 FM160725 FM160726 FM160727 FM160728 FM160729 FM160730 FM160731 FM160732 U85275 U85304 U11891 U11892 U11895 U11896 U11897 U11898 U11899 U11900 U11902 U11903 U11906 U11907 U11907 U11908 U11909 U11917 U11918 U85276 U85277 U85279 U85284 U85288 U85290 U85292 U85293 U85294 U85295 U85296 U85297 U85297 U85298 U85302
